# Physical activity in the treatment-resistant depression and non-remitted depression: a systematic review of randomized controlled trials

**DOI:** 10.12688/f1000research.140970.3

**Published:** 2024-08-27

**Authors:** José Etxaniz-Oses, Nagore Iriarte-Yoller, Mikel Tous-Espelosin, Sara Maldonado-Martin

**Affiliations:** 1 PHYSICAL EDUCATION AND SPORT, UNIVERSITY OF THE BASQUE COUNTRY (UPV/EHU), VITORIA-GASTEIZ, BASQUE COUNTRY, 01007, Spain; 2 Araba Mental Health Network, Osakidetza Basque Health Service, VITORIA-GASTEIZ, BASQUE COUNTRY, 01006, Spain

**Keywords:** Major depression, Exercise, Mental Disorder, Physical Activity, Remission

## Abstract

**Background:**

The objective of this systematic review was to analyze the effects of the physical activity (PA) intervention as an adjuvant strategy to pharmacological treatment in people with treatment-resistant depression (TRD) and non-remitted depression (NRD).

**Methods:**

A search strategy was realized from five databases: PubMed, Cochrane Central Register of Controlled Trials, Scopus, SPORTDiscus, and Web of Science. Eleven articles attained the inclusion criteria. The Physiotherapy Evidence Database and Oxford’s Evidence Levels were used to classify the quality appraisal.

**Results:**

The more significant outcome for this analysis was the improvement of depression by PA or exercise in TRD and NRD. According to the FITT (Frequency, Intensity, Time, and Type) principle, there was some variability in the PA intervention, and except for one article, they all were classified as excellent in terms of quality description.

**Conclusions:**

This review highlights the potential of PA intervention as an adjuvant program to improve different traits of TRD and NRD. The remission of depression seems to be higher after PA intervention, showing improvements in quality of life, sleep quality, executive function, and vitality.

## Introduction

Major depressive disorder (MDD) is one of the most prevalent mental illnesses in the world, as well as one of the most disabling ( Gutiérrez-Rojas *et al*., 2020). Considered a multifactorial disease, MDD has a difficult physiopathology ( Cichon *et al*., 2009). Besides antidepressant symptoms, the population with MDD presents toxic habits, such as smoking, alcohol intake, sedentarism, and physical inactivity, which leads to the appearance of different diseases ( *i.e.,* ictus, diabetes, cephalea, obesity, or coronary heart disease) ( Gutiérrez-Rojas *et al*., 2020). Adding to that, around 60% of people with MDD have a moderate remission; the remainder accomplish the treatment-resistant depression (TRD) or non-remitted depression (NRD) criteria ( Nemeroff, 2007). Although the definitions of TRD and NRD are unclear, in the two cases, the person who receives the pharmacological treatment does not have a remission, with a poor or unsatisfactory response to at least two adequate ( *i.e.,* optimal dosage and duration) different classes of antidepressants. However, previous research studies have demonstrated a lack of consensus criteria in defining TRD ( Harbi, 2012). On the other hand, NRD refers to patients’ reported partial response as defined by a screening Hamilton Depression Rating Scale (HDRS) score of ≥ 14 ( Trivedi *et al*., 2006). The treatment for NRD and TRD consists of first and second-generation antidepressants acting over cerebral synapsis, so there is increased bioavailability of amines (serotonin, noradrenaline, and dopamine) ( Cichon *et al*., 2009). Therefore, in this group of patients, a combination of different pharmacological therapy with antidepressant variety is not sufficient, and they could also need coadjuvant strategies such as physical activity (PA, *i.e.,* any movement that works your muscles and requires more energy than resting) and exercise ( *i.e.,* a subset of PA that is planned, structured, and repetitive with a final or an intermediate objective of improvement or maintenance of physical fitness). Thus, in the latest PA guidelines by the World Health Organization, 150-300 minutes of moderate and/or 75-150 minutes of high-intensity aerobic PA, in conjunction with two days of resistance training recommendations, have been included for people with mental disorders ( Bull *et al*., 2020).

A recent systematic review and network meta-analysis has stated that different kinds of PA and exercise ( *i.e.,* walking, strength training, mixed aerobic exercise, tai chi) are effective treatments for depression with proportional effects to the intensity prescribed ( *i.e.,* the higher the intensity, the greater the beneficial effect) ( Noetel et al., 2024). In this sense, in people with TRD and NRD, exercise was associated with a moderate improvement effect over depressive symptoms ( Carta *et al*., 2008; Harbi, 2012; Krogh *et al*., 2017; Mather *et al*., 2002; Mota-Pereira *et al*., 2011). However, no systematic review has been conducted so far.

Therefore, taking into account the recommendations above, the controversial outcomes, and the different exercise protocols related to the FITT principle ( *i.e.,* frequency, intensity, time, and type of exercise) in people with TRD and NRD, the purpose of this systematic review was to analyze the effects of PA intervention as an adjunct strategy to pharmacological treatment in people with TRD and NRD.

## Methods

The systematic review protocol has been recorded in the PROSPERO database (CRD42022298347). It follows the recommendations suggested by the Preferred Reporting Items for Systematic Review and Meta-analyses ( Page *et al.*, 2021) (see *Extended data*, ( Etxaniz-Oses *et al*., 2023)).

### Identification, selection, and eligibility criteria of studies

For the assessment and selection of the scientific articles, the following PICOS (Participant, Intervention, Comparator, Outcome, Study design) question was utilized:

(1) **P**opulation: TRD and NRD population over 18 years of age. Treatment-resistant depression refers to inadequate response to at least two antidepressants of adequate doses and duration ( Harbi, 2012). Non-remitted depression refers to patients’ reported partial response as defined by a screening Hamilton Depression Rating Scale (HDRS) score of ≥ 14 ( Trivedi *et al*., 2006). Studies including only men, only women, or both sexes were considered.
(2) **I**ntervention: any type of PA or exercise intervention that includes supervised exercise, online sessions, unsupervised activities, or recommendations.
(3) **C**omparator: intra-group (pre- vs. post-intervention) and inter-group (control vs. exercise intervention). Control participants were individuals with either TRD or NRD who did not undergo the PA intervention and continued with their usual treatment.
(4) **O**utcome: depressive symptoms. Depressive symptoms and any symptoms that indirectly affect these conditions (including physical, physiological, and quality of life parameters).
(5) **S**tudy design: randomized controlled trials (RCT) and randomized trials.

Two authors (S.M-M and J.E-O) were responsible for retrieving selected articles having in mind the following inclusion criteria: (a) Treatment-resistant or non-remitted depression, (b) physical activity or exercise, (c) clinical trials or experimental trials, (d) studies published in English, (e) scientific studies but not such as a book, magazines, online websites, reports, guidelines or recommendations or thesis. Scientific studies were excluded if they did not accomplish the eligibility PICOS questions. The following exclusion criteria were: (a) suffered depression and responded to treatment, (b) included PA interventions combined with other strategies, (c) the intervention was not adaptations of PA (d) had less than 18 of age, (e) there was not a comparison between pre-and post-intervention, and (f) it was not an RCT.

### Data sources and search strategy

A systematic literature search was conducted in June 2024 using the following online databases: PubMed, Cochrane Central Register of Controlled Trials, Scopus, SPORTDiscus, and Web of Science. The search was developed by synonyms of treatment resistant and physical activity or exercise and was performed by the followings search strategies: In PubMed (“major depression” OR “depression disorder” OR “depressive disorder” OR “treatment resistant depression” OR “non-remitted major depressive” OR “poorly responsive depressive” OR “treatment-resistant patients” OR “treatment-resistant major depressive”) AND (“physical activity” OR exercise); in Cochrane, Web of Science and SPORTDiscus “major depression” or “depression disorder” or “depressive disorder” or “treatment resistant depression” or “nonremitted major depressive” or “poorly responsive depressive” or “treatment-resistant patients” or “treatment-resistant major depressive”) and (“physical activity” or exercise); and in Scopus “major depression” OR “depression disorder” OR “depressive disorder” OR “treatment resistant depression” OR “nonremitted major depressive” OR “poorly responsive depressive” OR “treatment-resistant patients” OR “treatment-resistant major depressive” AND ““physical AND activity” OR “exercise””.

### Data screening and extraction

After duplicates were removed, the articles were revised by two independent authors (S.M-M and J.E-O) according to the title and abstract by the search strategy. After excluding studies based on title and abstract, any articles that remained questionable were analyzed more thoroughly by reading the full text. The chosen articles were read and assessed using eligibility criteria. In case of discrepancies, a consensus would be reached among the authors. The following articles were revised using Rayyan Intelligent Systematic Review Software: the software was developed specifically to expedite the initial screening of abstracts and titles using a semi-automation process. Likewise, in the publications, details of the investigation appear to show the screening by eligibility criteria.

### Outcome parameters

The more significant outcome for this analysis was the improvement of depression by PA or exercise in TRD and NRD.

### Quality appraisal

For assessing included scientific articles, the Physiotherapy Evidence Database (PEDro) scale ( de Morton, 2009) and Oxford’s Evidence Levels were used ( Howick *et al*., 2009). The PEDro scale rates RCT on a scale from 0 (low quality) to 11 (high quality) related to scientific rigor ( de Morton, 2009). Each measure is rated “yes” or “no”. Given that the assessors are rarely blinded and that it is impossible to blind the participants and investigators in supervised PA interventions, the items related to blinding (5–7) were removed from the scale. For this reason, the maximum result on the modified PEDro 8-point scale was 7 (highest score), as the first item is not included in the total score. The qualitative ratings were adjusted to those used in previous exercise-related systematic reviews as follows: 6–7 = “excellent”; 5 = “good”; 4 = “moderate”; and 0–3 = “poor”. Oxford’s Evidence levels range from 1 to 5, with 1a being systematic reviews of high-quality RCT, 1b individual RCT with a narrow confidence interval, 2a systematic review of cohort studies, 2b individual cohort study, 3a systematic review of a case-control study, 3b individual case-control study, 4 case-series, and 5 being expert opinions ( Howick *et al*., 2009). Two researchers (S.M-M and J.E-O) inspected the methodology of each study independently. Disagreements regarding the appraisal of methodological quality were resolved through discussion between the reviewers.

## Results

### Selection of studies

The database searches identified 10777 references (see *Extended data*, ( Etxaniz-Oses *et al*., 2023)). 3189 from PubMed, 1219 in Cochrane, 5698 in Web of Science, 567 in Scopus, and 104 in SPORTDiscus. After eliminating duplicates (n=3973) a total of 6804 articles were removed by title, abstract, study design, population, or type of intervention. Consequently, 20 articles were assessed for eligibility, and the assessment was done in full text. Finally, 11 articles were included in the systematic review. These were published between 2002 and 2017, showing the importance of PA in TRD and NRD (Supplementary data).

### Principal results and characteristics of studies

Physical activity as a predictor of various factors of mental illness as well as producing benefits in depression was the highlight of the articles ( Carta *et al*., 2008; Greer *et al*., 2014, 2016; Mather *et al*., 2002; Mota-Pereira *et al*., 2011; Rethorst *et al*., 2013a, 2013b, 2016; Toups *et al*., 2011, 2017; Trivedi, Greer, Blair, Church, Carmody, Grannemann, Galper, Dunn, Earnest, Sunderajan, & Henley, 2011).

The supplemental data included the main characteristics of the accepted studies in this systematic review. First, it is important to highlight that eight of the 11 chosen articles were from the same research project in the TRD or NRD population (n=275). Therefore, no variability was seen in the population and study design ( *i.e.,* two groups comparing different volumes of exercise doses) ( Greer *et al*., 2014, 2016; Rethorst *et al*., 2013a, 2013b, 2016; Toups *et al*., 2011, 2017; Trivedi *et al*., 2011). Nevertheless, the other three articles were not in the same research project ( Carta *et al*., 2008; Mather *et al*., 2002; Mota-Pereira *et al*., 2011). Concerning the type of PA assessed, while two articles conducted a novelty concurrent exercise intervention ( *i.e.,* including endurance and resistance training in the same session) ( Carta *et al*., 2008; Mather *et al*., 2002), a endurance training approach was observed in the other articles’ intervention, such as only aerobic continuous exercise (treadmill or cycle-ergometer) ( Greer *et al*., 2014, 2016; Rethorst *et al*., 2013a, 2013b, 2016; Toups *et al*., 2011, 2017; Trivedi *et al*., 2011), or weight-bearing strengthening exercise ( Mota-Pereira *et al*., 2011). None of the control groups performed any particular exercise, but pharmacological treatment ( Carta *et al*., 2008) or health-educational talks ( Mather *et al*., 2002).

In terms of the weekly frequency and time (volume) of PA, variability was shown in the studied articles (see *Extended data*, ( Etxaniz-Oses *et al*., 2023)). First, there was only one intervention five days per week ( Mota-Pereira *et al*., 2011), while the rest of the articles were performed two days per week. Second, the duration of the session ranges lasting from 45 to 60 minutes. In addition, the period of PA or exercise intervention to analyze the changes ranged from 10 ( Mather *et al*., 2002) to 12 weeks ( Greer *et al*., 2014, 2016; Mota-Pereira *et al*., 2011; Rethorst *et al*., 2013a, 2013b, 2016; Toups *et al*., 2011, 2017; Trivedi *et al*., 2011); but on the other hand, there was one article with a period of eight months of assessment ( Carta *et al*., 2008), and only one study presented a follow-up assessment after 24 weeks ( Mather *et al*., 2002). All the reviewed studies used moderate intensities for exercise and continuous endurance training controlled by a heart rate monitor ( Carta *et al*., 2008; Greer *et al*., 2014, 2016; Mota-Pereira *et al*., 2011; Rethorst *et al*., 2013a, 2013b, 2016; Toups *et al*., 2011, 2017) and rate of perceived exertion ( Mota-Pereira *et al*., 2011). However, in two studies, the intensity was not mentioned ( Carta *et al*., 2008; Mather *et al*., 2002), and another survey carried out endurance training on the treadmill with an absolute exercise intensity of 5 km/h ( Mota-Pereira *et al*., 2011). In this sense, the rest of the articles performed two different exercise doses ( *i.e.,* low dose: 4 kcal/kg/week (KKW) equivalent to 75 min/week, and high dose: 16 KKW equivalent to 210 min/week ( Greer *et al*., 2014, 2016; Rethorst *et al*., 2013a, 2013b, 2016; Toups *et al*., 2011, 2017; Trivedi *et al*., 2011).

Overall, after PA intervention, in all but one of the reviewed articles ( Rethorst *et al*., 2016), depression symptoms and functioning parameters improved, both in the TRD and NRD. The Hamilton Rating Scale for Depression (HRSD), Inventory of Depressive Symptoms-Clinical rated (IDS-C), Clinical Global Impression (CGI), and Geriatric Depression Scale (GDS), were the most widely used scales for depression screening ( Carta *et al*., 2008; Mather *et al*., 2002; Mota-Pereira *et al*., 2011; Rethorst *et al*., 2013a, 2013b). In addition, the quality of life assessed with different instruments such as SF-36 and The World Health Organization Quality of Life (WHOQOL) also improved significantly ( Greer *et al*., 2016). Further, sleep quality ( Rethorst *et al*., 2013a), pro-inflammatory levels ( Rethorst *et al*., 2013b), and some biomarkers of depression ( Toups *et al*., 2011) showed better values after PA intervention. In addition, executive function and working memory were improved depending on the exercise volume. Still, psychomotor speed, attention, visual memory, spatial planning, instrumental and expressive roll, and cognitive function improved irrespective of the type or dose of exercise participants undertook ( Greer *et al*., 2014, 2016). The remission of the participants also improved and was achieved in response to antidepressants with the PA as an adjuvant program on the different traits of TRD and NRD. Thus, in one of the RCTs, it was shown that 29.5% of the participants achieved remission ( Trivedi *et al*., 2011), and the remission was analyzed with different scales, such as HRSD, IDS-C, CGI, and GDS ( Mather *et al*., 2002; Mota-Pereira *et al*., 2011).

Although we understand that the meta-analysis of the results of the different studies may provide objective global information on specific variables, according to the chosen articles, our data are insufficient to perform the statistical procedure required for meta-analysis. Further, the diversity of the primary outcomes makes it even more difficult to combine data derived from the different studies. In this sense, a systematic review allows us to include those variables related to “what interests us” that cannot be meta-analyzed due to insufficient cohorts or test statistics.

Table 1 summarizes the PEDro scale and Oxford’s Evidence levels for the included articles in this systematic review. All the articles but one ( Carta *et al*., 2008), were classified as excellent. Inadequate concealed allocation to groups and the lack of similarity in the comparison groups at baseline were the methodological limitation in the article considered a “good” quality description. All papers were RCT, and for this reason, Oxford’s Evidence levels were 1b. All studies performed a comparison of both intra-group (pre- and post-PA-intervention) and inter-group (exercise vs. control group) when it was presented. Further, inclusion and exclusion criteria were defined, and blinding eligibility was presented.

**Table 1.** Assessment of the PEDro scale and Oxford’s Evidence levels of the included scientific articles that analyzed TRD and NRD after PA intervention.

PEDro ratings										Oxford’s Evidence Levels
	1	2	3	4	5	6	7	8	Total	
References										
( Mota-Pereira *et al.*, 2011)	Y	Y	Y	Y	Y	Y	Y	Y	7	1b
( Mather *et al.*, 2002)	Y	Y	Y	Y	Y	Y	Y	Y	7	1b
( Trivedi *et al.*, 2011)	Y	Y	Y	Y	Y	Y	Y	Y	7	1b
( Greer *et al.*, 2014)	Y	Y	Y	Y	Y	Y	Y	Y	7	1b
( Greer *et al.*, 2016)	Y	Y	Y	Y	Y	Y	Y	Y	7	1b
( Carta *et al.*, 2008)	Y	Y	N	N	Y	Y	Y	Y	5	1b
( Rethorst *et al.*, 2016)	Y	Y	Y	Y	Y	Y	Y	Y	7	1b
( Rethorst *et al.*, 2013a)	Y	Y	Y	Y	Y	Y	Y	Y	7	1b
( Rethorst *et al.*, 2013b)	Y	Y	Y	Y	Y	Y	Y	Y	7	1b
( Toups *et al.*, 2011)	Y	Y	Y	Y	Y	Y	Y	Y	7	1b
( Toups *et al.*, 2017)	Y	Y	Y	Y	Y	Y	Y	Y	7	1b

Abbreviations: N, no; NRD, non-remitted depression; PA, physical activity: TRD, treatment resistant depression; Y, yes.

(1) Eligibility criteria were specified. (2) Participants were randomized groups. (3) Allocation was concealed. (4) The groups were similar at baseline regarding the most important prognostic indicators. (5) Measures of at least one key outcome were obtained from more than 85% of participants initially allocated to groups. (6) All participants from whom outcome measures were available received the treatment or control condition as allocated or, where this was not the case, data for at least one key outcome was analyzed by “intention to treat”. (7) The results of between-group statistical comparisons are reported for at least one key outcome. (8) The study provides both point measures and measures of variability for at least one key outcome.

## Discussion

The objective of this study was to investigate the effects of PA intervention in TRD and NRD, describing the different parameters such as psychological, physical, and mental function and quality of life ( Carta *et al*., 2008; Greer *et al*., 2014, 2016; Mather *et al*., 2002; Mota-Pereira *et al*., 2011; Rethorst *et al*., 2013a, 2013b, 2016; Toups *et al*., 2011, 2017; Trivedi *et al*., 2011). The review determined 11 articles and identified that 1) PA intervention as an adjuvant program could improve different traits of TRD and NRD, 2) the remission of depression seems to be higher after PA intervention, also showing improvements in quality of life, sleep quality, executive function, or vitality, and 3) according to the FITT (Frequency, Intensity, Time, and Type) principle, there was some variability in the PA intervention, and except for one article ( Carta *et al*., 2008), they all were classified as excellent in terms of quality description.

A previous meta-analysis has found an interesting association between PA and incident depression, even below the public health recommendations, with an additional benefit when minimum recommendations are met ( Pearce *et al*., 2022). Thus, in the present systematic review, even though different types of intervention ( *i.e.,* concurrent or endurance training) were performed in the reviewed articles, the depression decreased via HRSD ( Mather *et al*., 2002; Mota-Pereira *et al*., 2011), and the quality of life increased ( Carta *et al*., 2008; Greer *et al*., 2016), irrespective of PA type. Concerning only resistance intervention in patients with depression, previous studies have compared aerobic *vs.* nonaerobic exercise training and observed that both groups reduced depression scores, but with no significant difference between groups ( Doyne *et al*., 1987; Martinsen *et al*., 1989). Taking into account all those above and the recent World Health Organization PA guidelines for people with chronic conditions ( Bull *et al*., 2020), it might be a good idea to include both resistance and endurance training in the adjuvant PA programs for TRD and NRD population, as it might result in better outcomes as seen in other populations ( Silva *et al*., 2015; Stanton & Reaburn, 2013). In this sense, the prefrontal cortex, anterior cingulate cortex, hippocampus, and corpus callosum emerge as possible neural markers that benefit from exercise in people with depression ( Gujral *et al*., 2017). It is time to confirm that during exercise, skeletal muscle releases valuable molecules that activate signals from skeletal muscle to different tissues, including the brain, highlighting that inactivity and loss of muscle mass may render the brain vulnerable to neurological dysfunction and disease ( Delezie & Handschin, 2018; Isaac *et al*., 2021). Regarding the frequency of weekly sessions, interventions ranged from two to five sessions, but the most frequently used was 2.6 sessions per week. This information was not observed in other systematic reviews, which noted that three sessions had the highest weekly frequency ( Perraton *et al.*, 2010; Stanton & Happell, 2013). Our results could be associated with TRD and NRD patients’ poorer physical condition and chronicity over time compared to those patients with a diagnosis of only depression. The duration of the interventions in the articles was between 10 and 12 weeks in almost all of them ( Greer *et al*., 2014, 2016; Mather *et al*., 2002; Mota-Pereira *et al*., 2011; Rethorst *et al*., 2013a, 2013b, 2016; Toups *et al*., 2011, 2017; Trivedi *et al*., 2011). However, another article used a more extended intervention with an eight-month intervention ( Carta *et al*., 2008). Other research on patients with depression used interventions for more than eight weeks ( Perraton e *t al.*, 2010). Yet, it may be that an intervention with a duration of 10-12 weeks would be sufficient to improve depression in TRD and NRD. Regarding the intensity of exercise, in the current study, all the reviewed articles performed moderate-intensity training interventions ( Carta *et al*., 2008; Greer *et al*., 2014, 2016; Mather *et al*., 2002; Mota-Pereira *et al*., 2011; Rethorst *et al*., 2013a, 2013b, 2016; Toups *et al*., 2011, 2017; Trivedi *et al*., 2011), with no studies including high-intensity training. Thus, recent investigations promote the relevance of exercise intensity and modality ( *i.e.,* high-intensity interval training) to induce a higher metabolic myokine effect on brain health ( Calverley *et al*., 2020; Hashimoto *et al*., 2021).

Three articles carried out a comparison between the control (no exercise intervention) and experimental group ( Carta *et al*., 2008; Mather *et al*., 2002; Mota-Pereira *et al*., 2011); the rest of the articles used a comparison with different doses of exercise ( Greer *et al*., 2014, 2016; Rethorst *et al*., 2013a, 2013b, 2016; Toups *et al*., 2011, 2017; Trivedi *et al*., 2011). In the articles that compared the control and experimental groups, the experimental groups observed better results than the control groups. In the same way, the groups that performed a high dose of exercise improved more than a low dose of exercise.

The present systematic review of RCTs analyzing the effects of PA intervention in patients with TRD and NRD determined the benefits of PA not only in the decrease of the depression symptoms but also in other variables related to the quality of life, such as sleep quality, executive and cognitive function, and memory. Therefore, regarding clinical practice and considering the results and the quality of the reviews, PA and exercise may be an efficient coadjuvant treatment for a patient with TRD or NRD, and it should be recommended. However, there is a complex interplay in the type of exercise program or intervention intensity; hence, there is a need for further investigation due to the limited evidence-based exercise intervention.

## Limitations and strengths

The most important limitation is that eight of the 11 studies are from the same project. Even so, it is essential to include them because they show different variables that may help to identify TRD or NRD. However, this is the first review to be carried out with these two populations in relation to exercise, and we have observed the lack of interventions in these two populations.

## Data Availability

All data underlying the results are available as part of the article and no additional source data are required. Zenodo: Physical activity in the treatment-resistance depression and non-remitted depression: a systematic review of randomized controlled trials, https://doi.org/10.5281/zenodo.10044119 ( Etxaniz-Oses *et al*., 2023). This project contains the following extended data:
- Extended data-supplementary data. Main characteristic of studies that analysed resistant treatment or non-remitted depression and physical activity - Flow diagram-supplementary data. Flowchart of the systematic review following the rules of PRISMA Zenodo: PRISMA checklist for ‘Physical activity in the treatment-resistance depression and non-remitted depression: a systematic review of randomized controlled trials’, https://doi.org/10.5281/zenodo.10044119 ( Etxaniz-Oses *et al*., 2023). Data are available under the terms of the Creative Commons Zero “No rights reserved” data waiver (CC0 1.0 Public domain dedication).
