# Physical activity in the treatment-resistant depression and non-remitted depression: a systematic review of randomized controlled trials

**DOI:** 10.12688/f1000research.140970.7

**Published:** 2026-01-16

**Authors:** José Etxaniz-Oses, Nagore Iriarte-Yoller, Mikel Tous-Espelosin, Sara Maldonado-Martin

**Affiliations:** 1 FACULTY OF PHYSICAL EDUCATION AND SPORT, UNIVERSITY OF THE BASQUE COUNTRY (UPV/EHU), VITORIA-GASTEIZ, BASQUE COUNTRY, 01007, Spain; 2 Araba Mental Health Network, Osakidetza Basque Health Service, VITORIA-GASTEIZ, BASQUE COUNTRY, 01006, Spain

**Keywords:** Major depression, Exercise, Mental Disorder, Physical Activity, Remission

## Abstract

**Background:**

The objective of this systematic review was to analyze the effects of physical activity (PA) intervention as an adjuvant strategy to pharmacological treatment in people with treatment-resistant depression (TRD) and non-remitted depression (NRD).

**Methods:**

A search strategy was conducted from five databases: PubMed, Cochrane Central Register of Controlled Trials, Scopus, SPORTDiscus, and Web of Science. The Physiotherapy Evidence Database and Oxford’s Evidence Levels were used to classify the quality appraisal.

**Results:**

Of the 10777 records, 11 randomized controlled studies met the inclusion criteria. The main outcome for this analysis was the effect of physical activity (PA) or exercise on depressive symptoms in people with TRD and NRD diagnosis. According to the FITT (Frequency, Intensity, Time, and Type) principle, there was some variability in the PA intervention. However, except for one article, all were classified as excellent in terms of quality description.

**Conclusions:**

This review highlights the potential of PA intervention as an adjuvant program to improve various traits in TRD and NRD. The remission of depression seems to be higher after PA intervention, showing improvements in quality of life, sleep quality, executive function, and vitality.

## Introduction

Major depressive disorder (MDD) is one of the most prevalent mental illnesses in the world, which may lead to disability ( Gutiérrez-Rojas *et al*., 2020). Considered a multifactorial disease, MDD has a complex physiopathology ( Cichon *et al*., 2009). Besides antidepressant symptoms, the population with MDD presents toxic habits, such as smoking, alcohol intake, sedentarism, and physical inactivity, which leads to the appearance of different diseases ( *i.e.,* stroke, diabetes, cephalea, obesity, or coronary heart disease) ( Gutiérrez-Rojas *et al*., 2020). Adding to that, around 60% of people with MDD have a moderate remission; the remainder accomplish the treatment-resistant depression (TRD) or non-remitted depression (NRD) criteria ( Nemeroff, 2007). Although the definitions of TRD and NRD are unclear, in both cases the person receiving pharmacological treatment does not have remission, with a poor or unsatisfactory response to at least two adequate ( *i.e.,* optimal dosage and duration) different classes of antidepressants. However, previous research has shown a lack of consensus criteria in defining TRD ( Harbi, 2012). On the other hand, NRD refers to patients’ reported partial response, defined as a screening Hamilton Depression Rating Scale (HDRS) score of ≥ 14 ( Trivedi *et al*., 2006). The treatment for NRD and TRD consists of first- and second-generation antidepressants that act at the cerebral synapse, thereby increasing the bioavailability of amines (serotonin, noradrenaline, and dopamine) ( Cichon *et al*., 2009). Therefore, in this group of patients, a combination of different pharmacological therapy with antidepressant variety is not sufficient. Hence, they could also need adjuvant strategies such as physical activity (PA, *i.e.,* any movement that works your muscles and requires more energy than resting) and exercise ( *i.e.,* a subset of PA that is planned, structured, and repetitive with a final or an intermediate objective of improvement or maintenance of physical fitness). Thus, in the latest PA guidelines by the World Health Organization, 150-300 minutes of moderate and/or 75-150 minutes of high-intensity aerobic PA, in conjunction with two days of resistance training recommendations, have been included for people with mental disorders ( Bull *et al*., 2020).

A recent systematic review and network meta-analysis has stated that different kinds of PA and exercise ( *i.e.,* walking, strength training, mixed aerobic exercise, tai chi) are effective treatments for depression with proportional effects to the intensity prescribed ( *i.e.,* the higher the intensity, the greater the beneficial effect) ( Noetel et al., 2024). Although the beneficial effect of exercise on depression in general has been documented, to date, no systematic review has been conducted focused explicitly on populations with TRD and NRD. This review provides structured evidence on the effectiveness of PA as an adjuvant strategy in these clinically complex subgroups. In this sense, in people with TRD and NRD, exercise was associated with a moderate improvement in depressive symptoms ( Carta *et al*., 2008; Harbi, 2012; Krogh *et al*., 2017; Mather *et al*., 2002; Mota-Pereira *et al*., 2011). However, no systematic review has been conducted so far.

Therefore, taking into account the recommendations above, the controversial outcomes, and the different exercise protocols related to the FITT principle ( *i.e.,* Frequency, Intensity, Time, and Type of exercise) in people with TRD and NRD, the purpose of this systematic review was to analyze the effects of PA intervention as an adjunct strategy to pharmacological treatment in people with TRD and NRD. Thus, it is hypothesized that also in people with TRD and NRD, PA and exercise will be an effective adjuvant program to pharmacological treatment.

## Methods

The systematic review protocol has been recorded in the PROSPERO database (CRD42022298347). It follows the recommendations suggested by the Preferred Reporting Items for Systematic Review and Meta-analyses ( Page *et al.*, 2021) (see *Extended data*, ( Etxaniz-Oses *et al*., 2023)).

### Identification, selection, and eligibility criteria of studies

For the assessment and selection of the scientific articles, the following PICOS (Participant, Intervention, Comparator, Outcome, Study design) question was utilized:

(1) **P**opulation. TRD and NRD population over 18 years of age. Treatment-resistant depression refers to inadequate response to at least two antidepressants of adequate doses and duration ( Harbi, 2012). Non-remitted depression refers to patients’ reported partial response as defined by a screening Hamilton Depression Rating Scale (HDRS) score of ≥ 14 ( Trivedi *et al*., 2006). Studies including only men, only women, or both sexes were considered.
(2) **I**ntervention. Any type of PA or exercise intervention that includes supervised exercise, online sessions, unsupervised activities, or recommendations. Interventions involving sports were not considered or included in the search strategy.
(3) **C**omparator. The following control groups were taken into account: 1) Treatment as Usual: participants who continue with their standard pharmacological treatment and usual clinical care without any structured PA component. 2) Active Control: groups that receive an alternative intervention unrelated to exercise (e.g., educational sessions on health, relaxation therapy, or low-intensity psychological support) designed to control for the effects of attention, time, and social interaction. 3) No-Intervention/Wait-list Control: groups that do not receive any additional intervention or structured activity during the study period, although they may continue with their baseline pharmacological treatment. This comparison assesses the specific effect of exercise versus the natural progression of the condition.
(4) **O**utcome. Depressive symptoms and any symptoms that indirectly affect these conditions (including physical, physiological, and quality of life parameters).
(5) **S**tudy design: randomized controlled trials (RCT) and other randomized intervention trials.

Two authors (S.M-M and J.E-O) were responsible for retrieving selected articles having in mind the following inclusion criteria: (a) Treatment-resistant or non-remitted depression, (b) physical activity or exercise, (c) clinical trials or experimental trials, (d) studies published in English, (e) scientific studies but not such as a book, magazines, online websites, reports, guidelines or recommendations or thesis. Scientific studies were excluded if they did not meet the eligibility criteria for the PICOS questions. The following exclusion criteria were: (a) suffered depression and responded to treatment, (b) included PA interventions combined with other strategies, (c) the intervention was not an adaptation of PA (d) had less than 18 of age, (e) there was not a comparison between pre-and post-intervention, and (f ) it was not an RCT. These comparator categories were predefined before study selection to reduce conceptual heterogeneity. This approach has been previously adopted in systematic reviews of exercise interventions, where participant and therapist blinding is inherently unfeasible.

### Data sources and search strategy

A systematic literature search was conducted in June 2024 using the following online databases: PubMed, Cochrane Central Register of Controlled Trials, Scopus, SPORTDiscus, and Web of Science. The search was developed by synonyms of treatment resistant and physical activity or exercise and was performed by the followings search strategies: In PubMed (“major depression” OR “depression disorder” OR “depressive disorder” OR “treatment resistant depression” OR “non-remitted major depressive” OR “poorly responsive depressive” OR “treatment-resistant patients” OR “treatment-resistant major depressive”) AND (“physical activity” OR exercise); in Cochrane, Web of Science and SPORTDiscus “major depression” or “depression disorder” or “depressive disorder” or “treatment resistant depression” or “nonremitted major depressive” or “poorly responsive depressive” or “treatment-resistant patients” or “treatment-resistant major depressive”) and (“physical activity” or exercise); and in Scopus “major depression” OR “depression disorder” OR “depressive disorder” OR “treatment resistant depression” OR “nonremitted major depressive” OR “poorly responsive depressive” OR “treatment-resistant patients” OR “treatment-resistant major depressive” AND ““physical AND activity” OR “exercise””.

### Data screening and extraction

After duplicates were removed, the articles were revised by two independent authors (S.M-M and J.E-O) based on the titles and abstracts generated by the search strategy. After this procedure, any article that remained questionable was analyzed more thoroughly by reading the full text. The chosen articles were read and assessed using eligibility criteria. In case of discrepancies, the authors would reach consensus. The following articles were revised using Rayyan Intelligent Systematic Review Software, which was developed specifically to expedite the initial screening of abstracts and titles using a semi-automated process. Likewise, in the publications, the details of the investigation appear to show that screening was conducted according to eligibility criteria.

### Outcome parameters

The more significant outcome for this analysis was the improvement of depression by PA or exercise in TRD and NRD.

### Quality appraisal

For assessing included scientific articles, the Physiotherapy Evidence Database (PEDro) scale ( de Morton, 2009) and Oxford’s Evidence Levels were used ( Howick *et al*., 2009). The PEDro scale rates RCT on a scale from 0 (low quality) to 11 (high quality) related to scientific rigor ( de Morton, 2009). Each measure is rated “yes” or “no”. Items related to blinding of participants and assessors were considered in the context of exercise-based interventions, where blinding is often not feasible. Therefore, these items were not used to penalize study quality scores, and results were interpreted accordingly. This approach is consistent with previous systematic reviews of exercise-related research. For this reason, the maximum score on the modified PEDro 8-point scale was 7 (the highest), since the first item is not included in the total score. The qualitative ratings were adjusted to those used in previous exercise-related systematic reviews as follows: 6–7 = “excellent”; 5 = “good”; 4 = “moderate”; and 0–3 = “poor”. Oxford’s Evidence levels range from 1 to 5, with 1a being systematic reviews of high-quality RCT, 1b individual RCT with a narrow confidence interval, 2a systematic review of cohort studies, 2b individual cohort study, 3a systematic review of a case-control study, 3b individual case-control study, 4 case-series, and 5 being expert opinions ( Howick *et al*., 2009). Two researchers (S.M-M and J.E-O) inspected the methodology of each study independently. Disagreements regarding the appraisal of methodological quality were resolved through discussion between the reviewers.

## Searching results

### Selection of studies

The database searches identified 10777 references (see *Extended data*, ( Etxaniz-Oses *et al*., 2023)). 3189 from PubMed, 1219 in Cochrane, 5698 in Web of Science, 567 in Scopus, and 104 in SPORTDiscus. After eliminating duplicates (n=3973), a total of 6804 articles were removed by title, abstract, study design, population, or type of intervention. Consequently, 20 articles were assessed for eligibility, and the assessment was done in full text. Finally, 11 articles were included in the systematic review. These were published between 2002 and 2017, showing the importance of PA in TRD and NRD (Supplementary data).

### Principal results and characteristics of studies

Physical activity as a predictor of various factors of mental illness, as well as producing benefits in depression was the highlight of the articles ( Carta *et al*., 2008; Greer *et al*., 2014, 2016; Mather *et al*., 2002; Mota-Pereira *et al*., 2011; Rethorst *et al*., 2013a, 2013b, 2016; Toups *et al*., 2011, 2017; Trivedi, Greer, Blair, Church, Carmody, Grannemann, Galper, Dunn, Earnest, Sunderajan, & Henley, 2011).

The supplemental data included the main characteristics of the accepted studies in this systematic review. First, it is important to highlight that eight of the 11 selected articles were from the same research project in the TRD or NRD population (n=275). Therefore, no variability was observed in the population and study design ( *i.e.,* two groups comparing different volumes of exercise doses) ( Greer *et al*., 2014, 2016; Rethorst *et al*., 2013a, 2013b, 2016; Toups *et al*., 2011, 2017; Trivedi *et al*., 2011). Nevertheless, the other three articles were not part of the same research project ( Carta *et al*., 2008; Mather *et al*., 2002; Mota-Pereira *et al*., 2011). Concerning the type of PA assessed, while two articles conducted a novelty concurrent exercise intervention ( *i.e.,* including endurance and resistance training in the same session) ( Carta *et al*., 2008; Mather *et al*., 2002), an endurance training approach was observed in the other articles’ intervention, such as only aerobic continuous exercise (treadmill or cycle-ergometer) ( Greer *et al*., 2014, 2016; Rethorst *et al*., 2013a, 2013b, 2016; Toups *et al*., 2011, 2017; Trivedi *et al*., 2011), or weight-bearing strengthening exercise ( Mota-Pereira *et al*., 2011). None of the control groups performed any particular exercise, but pharmacological treatment ( Carta *et al*., 2008) or health-educational talks ( Mather *et al*., 2002).

In terms of the weekly frequency and time (volume) of PA, variability was shown in the studied articles (see *Extended data*, ( Etxaniz-Oses *et al*., 2023)). First, there was only one intervention, five days per week ( Mota-Pereira *et al*., 2011), whereas the rest of the articles were performed two days per week. Second, the duration of the session ranges from 45 to 60 minutes. In addition, the period of PA or exercise intervention to analyze the changes ranged from 10 ( Mather *et al*., 2002) to 12 weeks ( Greer *et al*., 2014, 2016; Mota-Pereira *et al*., 2011; Rethorst *et al*., 2013a, 2013b, 2016; Toups *et al*., 2011, 2017; Trivedi *et al*., 2011); but on the other hand, there was one article with a period of eight months of assessment ( Carta *et al*., 2008), and only one study presented a follow-up assessment after 24 weeks ( Mather *et al*., 2002). All the reviewed studies used moderate intensities for exercise and continuous endurance training controlled by a heart rate monitor ( Carta *et al*., 2008; Greer *et al*., 2014, 2016; Mota-Pereira *et al*., 2011; Rethorst *et al*., 2013a, 2013b, 2016; Toups *et al*., 2011, 2017) and rate of perceived exertion ( Mota-Pereira *et al*., 2011). However, in two studies, the intensity was not mentioned ( Carta *et al*., 2008; Mather *et al*., 2002), and another survey reported endurance training on a treadmill with an absolute exercise intensity of 5 km/h ( Mota-Pereira *et al*., 2011). In this sense, the rest of the articles performed two different exercise doses ( *i.e.,* low dose: 4 kcal/kg/week (KKW) equivalent to 75 min/week, and high dose: 16 KKW equivalent to 210 min/week ( Greer *et al*., 2014, 2016; Rethorst *et al*., 2013a, 2013b, 2016; Toups *et al*., 2011, 2017; Trivedi *et al*., 2011).

Overall, after PA intervention, in all but one of the reviewed articles ( Rethorst *et al*., 2016), depression symptoms and functioning parameters improved, in both TRD and NRD. The Hamilton Rating Scale for Depression (HRSD), Inventory of Depressive Symptoms-Clinical rated (IDS-C), Clinical Global Impression (CGI), and Geriatric Depression Scale (GDS), were the most widely used scales for depression screening ( Carta *et al*., 2008; Mather *et al*., 2002; Mota-Pereira *et al*., 2011; Rethorst *et al*., 2013a, 2013b). In addition, quality of life, as assessed with instruments such as the SF-36 and the World Health Organization Quality of Life (WHOQOL) also improved significantly ( Greer *et al*., 2016). Further, sleep quality ( Rethorst *et al*., 2013a), pro-inflammatory levels ( Rethorst *et al*., 2013b), and some biomarkers of depression ( Toups *et al*., 2011) showed better values after PA intervention. In addition, executive function and working memory improved with increasing exercise volume. Still, psychomotor speed, attention, visual memory, spatial planning, instrumental and expressive roles, and cognitive function improved irrespective of the type or dose of exercise participants undertook ( Greer *et al*., 2014, 2016). The remission of the participants also improved and was achieved in response to antidepressants with the PA as an adjuvant program on the different traits of TRD and NRD. Thus, in one of the RCTs, it was shown that 29.5% of the participants achieved remission ( Trivedi *et al*., 2011), and the remission was analyzed with different scales, such as HRSD, IDS-C, CGI, and GDS ( Mather *et al*., 2002; Mota-Pereira *et al*., 2011).

A meta-analysis was not conducted because several studies did not report the statistical data required for quantitative synthesis (e.g., standard deviations or effect estimates for remission/response). For this reason, a meta-analysis can actually assess heterogeneity, which can be taken into consideration in the final quality of evidence and interpretation.

### Risk of bias

Table 1 summarizes the PEDro scale and Oxford’s Evidence levels for the included articles in this systematic review. All the articles except one ( Carta *et al*., 2008), were classified as excellent. Inadequate concealed allocation to groups and the lack of similarity in the comparison groups at baseline were the methodological limitations in the article, which was considered a “good” quality description. All papers were RCT, and for this reason, Oxford’s Evidence levels were 1b. All studies performed comparisons of both intra-group (pre- and post-PA-intervention) and inter-group (exercise vs. control group) when presented. Further, inclusion and exclusion criteria were defined, and blinding eligibility was presented.

**Table 1.** Assessment of the PEDro scale and Oxford’s Evidence levels of the included scientific articles that analyzed TRD and NRD after PA intervention.

PEDro ratings										Oxford’s Evidence Levels
	1	2	3	4	5	6	7	8	Total	
References										
( Mota-Pereira *et al.*, 2011)	Y	Y	Y	Y	Y	Y	Y	Y	7	1b
( Mather *et al.*, 2002)	Y	Y	Y	Y	Y	Y	Y	Y	7	1b
( Trivedi *et al.*, 2011)	Y	Y	Y	Y	Y	Y	Y	Y	7	1b
( Greer *et al.*, 2014)	Y	Y	Y	Y	Y	Y	Y	Y	7	1b
( Greer *et al.*, 2016)	Y	Y	Y	Y	Y	Y	Y	Y	7	1b
( Carta *et al.*, 2008)	Y	Y	N	N	Y	Y	Y	Y	5	1b
( Rethorst *et al.*, 2016)	Y	Y	Y	Y	Y	Y	Y	Y	7	1b
( Rethorst *et al.*, 2013a)	Y	Y	Y	Y	Y	Y	Y	Y	7	1b
( Rethorst *et al.*, 2013b)	Y	Y	Y	Y	Y	Y	Y	Y	7	1b
( Toups *et al.*, 2011)	Y	Y	Y	Y	Y	Y	Y	Y	7	1b
( Toups *et al.*, 2017)	Y	Y	Y	Y	Y	Y	Y	Y	7	1b

Abbreviations: N, no; NRD, non-remitted depression; PA, physical activity: TRD, treatment resistant depression; Y, yes.

(1) Eligibility criteria were specified. (2) Participants were randomized groups. (3) Allocation was concealed. (4) The groups were similar at baseline regarding the most important prognostic indicators. (5) Measures of at least one key outcome were obtained from more than 85% of participants initially allocated to groups. (6) All participants from whom outcome measures were available received the treatment or control condition as allocated or, where this was not the case, data for at least one key outcome was analyzed by “intention to treat”. (7) The results of between-group statistical comparisons are reported for at least one key outcome. (8) The study provides both point measures and measures of variability for at least one key outcome.

## Discussion

The objective of this study was to investigate the effects of PA intervention in TRD and NRD, describing the different parameters such as psychological, physical, and mental function and quality of life ( Carta *et al*., 2008; Greer *et al*., 2014, 2016; Mather *et al*., 2002; Mota-Pereira *et al*., 2011; Rethorst *et al*., 2013a, 2013b, 2016; Toups *et al*., 2011, 2017; Trivedi *et al*., 2011). The review determined 11 articles and identified that 1) PA intervention as an adjuvant program could improve different traits of TRD and NRD, 2) the remission of depression seems to be higher after PA intervention, also showing improvements in quality of life, sleep quality, executive function, or vitality, and 3) according to the FITT principle, there was some variability in the PA intervention, and except for one article ( Carta *et al*., 2008), they all were classified as excellent in terms of quality description.

A previous meta-analysis has found an interesting association between PA and incident depression, even below the public health recommendations, with an additional benefit when minimum recommendations were met ( Pearce *et al*., 2022). Thus, in the present systematic review, even though different types of intervention ( *i.e.,* concurrent or endurance training) were performed in the reviewed articles, the depression decreased via HRSD ( Mather *et al*., 2002; Mota-Pereira *et al*., 2011), and the quality of life increased ( Carta *et al*., 2008; Greer *et al*., 2016), irrespective of PA type. Concerning only resistance intervention in patients with depression, previous studies have compared aerobic *vs.* nonaerobic exercise training and observed that both groups reduced depression scores, but with no significant difference between groups ( Doyne *et al*., 1987; Martinsen *et al*., 1989). Taking into account all those above and the World Health Organization PA guidelines for people with chronic conditions ( Bull *et al*., 2020), these results suggest to include both resistance and endurance training in the adjuvant PA programs for TRD and NRD population, as it might result in better outcomes as seen in other populations ( Silva *et al*., 2015; Stanton & Reaburn, 2013). In this sense, the prefrontal cortex, anterior cingulate cortex, hippocampus, and corpus callosum emerge as possible neural markers that benefit from exercise in people with depression ( Gujral *et al*., 2017). These findings support the idea that during exercise, skeletal muscle releases valuable molecules that activate signals from skeletal muscle to different tissues, including the brain, highlighting that inactivity and loss of muscle mass may render the brain vulnerable to neurological dysfunction and disease ( Delezie & Handschin, 2018; Isaac *et al*., 2021). Regarding the frequency of weekly sessions, interventions ranged from two to five sessions, with the most frequently used being 2.6 sessions per week. This information was not observed in other systematic reviews, which reported that three sessions had the highest weekly frequency ( Perraton *et al.*, 2010; Stanton & Happell, 2013). Our results could be associated with TRD and NRD patients’ poorer physical condition and chronicity over time compared to those patients with a diagnosis of only depression. The duration of the interventions in the articles was between 10 and 12 weeks in almost all of them ( Greer *et al*., 2014, 2016; Mather *et al*., 2002; Mota-Pereira *et al*., 2011; Rethorst *et al*., 2013a, 2013b, 2016; Toups *et al*., 2011, 2017; Trivedi *et al*., 2011). However, another article used an extended eight-month intervention ( Carta *et al*., 2008). Other research on patients with depression used interventions for more than eight weeks ( Perraton e *t al.*, 2010). Yet, it may be that an intervention with a duration of 10-12 weeks would be sufficient to improve depression in TRD and NRD. Regarding the intensity of exercise, in the current study, all the reviewed articles performed moderate-intensity training interventions ( Carta *et al*., 2008; Greer *et al*., 2014, 2016; Mather *et al*., 2002; Mota-Pereira *et al*., 2011; Rethorst *et al*., 2013a, 2013b, 2016; Toups *et al*., 2011, 2017; Trivedi *et al*., 2011), with no studies including high-intensity training. Thus, recent investigations promote the relevance of exercise intensity and modality ( *i.e.,* high-intensity interval training) to induce a higher metabolic myokine effect on brain health ( Calverley *et al*., 2020; Hashimoto *et al*., 2021).

Three articles carried out a comparison between the control (no exercise intervention) and experimental group ( Carta *et al*., 2008; Mather *et al*., 2002; Mota-Pereira *et al*., 2011); the rest of the articles used a comparison with different doses of exercise ( Greer *et al*., 2014, 2016; Rethorst *et al*., 2013a, 2013b, 2016; Toups *et al*., 2011, 2017; Trivedi *et al*., 2011). In the articles that compared the control and experimental groups, the experimental groups observed better results than the control groups. In the same way, the groups that performed a high dose of exercise improved more than those that performed a low dose.

The present systematic review of RCTs analyzing the effects of PA intervention in patients with TRD and NRD determined the benefits of PA not only reducing depressive symptoms but also in other variables related to quality of life, such as sleep quality, executive and cognitive function, and memory. Therefore, in clinical practice, considering the results and quality of the reviews, PA and exercise may be an efficient adjuvant treatment for a patient with TRD or NRD, and should be recommended. However, there is a complex interplay between the type of exercise program and the intensity of the intervention; hence, further investigation is needed. Clinicians should consider structured exercise programs as part of multidisciplinary management in TRD and NRD.

## Limitations and strengths

The most important limitation is that eight of the 11 studies are from the same project. No attempt was made to retrieve data that could be used for a meta-analysis because the magnitude of the missing data was large. Even so, it is essential to include them because they show different variables that may help to identify TRD or NRD. However, this is the first review to examine these two populations regarding exercise, and we have observed a lack of interventions in both.

## Data Availability

All data underlying the results are available as part of the article and no additional source data are required. Zenodo: Physical activity in the treatment-resistant depression and non-remitted depression: a systematic review of randomized controlled trials, https://doi.org/10.5281/zenodo.10044119 ( Etxaniz-Oses *et al*., 2023). This project contains the following extended data:
- Extended data-supplementary data. Main characteristic of studies that analyzed resistant treatment or non-remitted depression and physical activity - Flow diagram-supplementary data. Flowchart of the systematic review following the rules of PRISMA Zenodo: PRISMA checklist for ‘Physical activity in the treatment-resistant depression and non-remitted depression: a systematic review of randomized controlled trials’, https://doi.org/10.5281/zenodo.10044119 ( Etxaniz-Oses *et al*., 2023). Data are available under the terms of the Creative Commons Zero “No rights reserved” data waiver (CC0 1.0 Public domain dedication).
