# Physical activity in the treatment-resistant depression and non-remitted depression: a systematic review of randomized controlled trials

**DOI:** 10.12688/f1000research.140970.8

**Published:** 2026-07-01

**Authors:** José Etxaniz-Oses, Nagore Iriarte-Yoller, Mikel Tous-Espelosin, Sara Maldonado-Martin

**Affiliations:** 1 FACULTY OF PHYSICAL EDUCATION AND SPORT, UNIVERSITY OF THE BASQUE COUNTRY (UPV/EHU), VITORIA-GASTEIZ, BASQUE COUNTRY, 01007, Spain; 2 Araba Mental Health Network, Osakidetza Basque Health Service, VITORIA-GASTEIZ, BASQUE COUNTRY, 01006, Spain

**Keywords:** Major depression, Exercise, Mental Disorder, Physical Activity, Remission

## Abstract

**Background:**

The objective of this systematic review was to analyze the effects of physical activity (PA) intervention as an adjuvant strategy to pharmacological treatment in people with treatment-resistant depression (TRD) and non-remitted depression (NRD).

**Methods:**

A search strategy was conducted from five databases: PubMed, Cochrane Central Register of Controlled Trials, Scopus, SPORTDiscus, and Web of Science. The Physiotherapy Evidence Database and Oxford’s Evidence Levels were used to classify the quality appraisal.

**Results:**

Of the 10777 records, 11 randomized controlled studies met the inclusion criteria, comprising 567 participants (n=275 from the TREAD project across eight sub-studies; n=292 from three independent Randomized control trials). The main outcome for this analysis was the effect of physical activity (PA) or exercise on depressive symptoms in people with TRD and NRD diagnosis. According to the FITT (Frequency, Intensity, Time, and Type) principle, there was some variability in the PA intervention. However, except for one article, all were classified as excellent in terms of quality description.

**Conclusions:**

Based on moderate-quality evidence from three independent RCTs, structured exercise programs may be considered as an adjunctive intervention for depressive symptoms in patients with TRD and NRD. Evidence for other outcomes (quality of life, sleep, cognition, biomarkers) is of low or very low quality, derived from single studies or non-independent TREAD sub-analyses. Future research should prioritize independent replication of secondary outcomes and pre-specified analyses of predictors of response. Further high-quality RCTs in this population are urgently needed to confirm these findings and establish optimal PA protocols.

## Introduction

Major depressive disorder (MDD) is one of the most prevalent mental illnesses in the world, which may lead to disability ( Gutiérrez-Rojas *et al*., 2020). Considered a multifactorial disease, MDD has a complex physiopathology ( Cichon *et al*., 2009). Adding to that, around 60% of people with MDD have a moderate remission; the remainder accomplish the treatment-resistant depression (TRD) or non-remitted depression (NRD) criteria ( Nemeroff, 2007). The definition of TRD remains unclear. It is generally used to describe individuals who do not achieve remission following pharmacological treatment and show a poor or unsatisfactory response to at least two antidepressants from different classes, administered at adequate doses and for an appropriate duration. However, in this sense, previous research has shown a lack of consensus criteria in defining TRD ( Harbi, 2012). Now, experts agree that TRD should be defined dimensionally (as a spectrum of severity) rather than just dichotomously (yes/no), as it better reflects the progression of illness ( Rybak *et al.*, 2021).

On the other hand, NRD is characterized by insufficient improvement that does not meet the established thresholds for remission. For its diagnosis, scales such as the Hamilton Depression Rating Scale (HDRS) are used ( Mendlewicz, 2008). Both the definition of TRD and that of NRD are problematic because they may suggest an artificial boundary; authors propose a personalized approach in which various factors (biological, psychological, sociocultural, lifestyle) are identified so that treatment can be tailored to address the same issue: the lack of remission ( Dodd *et al.*, 2021).

The treatment for NRD and TRD consists of first- and second-generation antidepressants that act at the cerebral synapse, thereby increasing the bioavailability of amines (serotonin, noradrenaline, and dopamine) ( Cichon *et al*., 2009). Besides antidepressant symptoms, the population with MDD presents toxic habits, such as smoking, alcohol intake, sedentarism, and physical inactivity, which leads to the appearance of different diseases ( *i.e.,* stroke, diabetes, cephalea, obesity, or coronary heart disease) and is no different from people with depression who do not experience remission ( Gutiérrez-Rojas *et al*., 2020). Patients with depression who do not achieve remission present unfavorable lifestyle patterns; therefore, an intervention targeting lifestyle modification may be warranted ( Kunugi, 2023). Therefore, in this group of patients, a combination of different pharmacological therapies with antidepressant variety is not sufficient. Hence, they could also need adjuvant strategies such as physical activity (PA, *i.e.,* any movement that works your muscles and requires more energy than resting) and exercise ( *i.e.,* a subset of PA that is planned, structured, and repetitive with a final or an intermediate objective of improvement or maintenance of physical fitness). Furthermore, exercise and PA can improve both symptoms of the disease and quality of life in this population ( Kunugi, 2023). Thus, in the latest PA guidelines by the World Health Organization, 150-300 minutes of moderate and/or 75-150 minutes of high-intensity aerobic PA, in conjunction with two days of resistance training, have been included for people with mental disorders ( Bull *et al*., 2020).

A recent systematic review and network meta-analysis has stated that different kinds of PA and exercise ( *i.e.,* walking, strength training, mixed aerobic exercise, tai chi) are effective treatments for depression with proportional effects to the intensity prescribed ( *i.e.,* the higher the intensity, the greater the beneficial effect) ( Noetel *et al.*, 2024). Chronic inflammation is commonly observed in people with depression. Exercise has been shown to exert anti-inflammatory effects, enhance dopaminergic transmission, counteract inflammation-induced alterations in the reward system, and improve mitochondrial metabolism ( Munro *et al.*, 2026). Exercise has the potential to ameliorate the mitochondrial dysfunction characteristic of TRD, promote neuroplasticity and neurogenesis, and decrease both oxidative stress and neuroinflammation ( Ramos-Jiménez *et al.*, 2025). Although such improvements could theoretically extend to individuals with TRD and NRD, the available evidence is limited. This review provides structured evidence on the effectiveness of PA as an adjuvant strategy in these clinically complex subgroups. In this sense, in people with TRD and NRD, exercise was associated with a moderate improvement in depressive symptoms ( Carta *et al*., 2008; Harbi, 2012; Krogh *et al*., 2017; Mather *et al*., 2002; Mota-Pereira *et al*., 2011). However, no systematic review has been conducted so far.

Therefore, taking into account the recommendations above, the controversial outcomes, and the different exercise protocols related to the FITT principle ( *i.e.,* Frequency, Intensity, Time, and Type of exercise) in people with TRD and NRD, the purpose of this systematic review was to analyze the effects of PA intervention as an adjunct strategy to pharmacological treatment in people with TRD and NRD. Thus, it is hypothesized that also in people with TRD and NRD, PA and exercise will be an effective adjuvant program to pharmacological treatment.

## Methods

The systematic review protocol has been recorded in the PROSPERO database (CRD42022298347). It follows the recommendations suggested by the Preferred Reporting Items for Systematic Review and Meta-analyses ( Page *et al.*, 2021) (see *Extended data*, ( Etxaniz-Oses *et al*., 2023)).

### Identification, selection, and eligibility criteria of studies

For the assessment and selection of the scientific articles, the following PICOS (Participant, Intervention, Comparator, Outcome, Study design) question was utilized:

(1) **P**opulation. TRD and NRD population over 18 years of age. Treatment-resistant depression refers to inadequate response to at least two antidepressants of adequate doses and duration ( Harbi, 2012). Non-remitted depression refers to patients’ reported partial response as defined by a screening Hamilton Depression Rating Scale (HDRS) score of ≥ 14 ( Trivedi *et al*., 2006). Studies including only men, only women, or both sexes were considered.
(2) **I**ntervention. Any type of PA or exercise intervention that includes supervised exercise, online sessions, unsupervised activities, or recommendations. Interventions involving sports were not considered or included in the search strategy.
(3) **C**omparator. The following control groups were taken into account: 1) Treatment as Usual: participants who continue with their standard pharmacological treatment and usual clinical care without any structured PA component. 2) Active Control: groups that receive an alternative intervention unrelated to exercise (e.g., educational sessions on health, relaxation therapy, or low-intensity psychological support) designed to control for the effects of attention, time, and social interaction. 3) No-Intervention/Wait-list Control: groups that do not receive any additional intervention or structured activity during the study period, although they may continue with their baseline pharmacological treatment. This comparison assesses the specific effect of exercise versus the natural progression of the condition.
(4) **O**utcome. Depressive symptoms and any symptoms that indirectly affect these conditions (including physical, physiological, and quality of life parameters).
(5) **S**tudy design: randomized controlled trials (RCT) and other randomized intervention trials.

Two authors (S.M-M and J.E-O) were responsible for retrieving selected articles having in mind the following inclusion criteria: (a) Treatment-resistant or non-remitted depression, (b) physical activity or exercise, (c) clinical trials or experimental trials, (d) studies published in English, (e) scientific studies but not such as a book, magazines, online websites, reports, guidelines or recommendations or thesis. Scientific studies were excluded if they did not meet the eligibility criteria for the PICOS questions. The following exclusion criteria were: (a) suffered depression and responded to treatment, (b) included PA interventions combined with other strategies, (c) the intervention was not an adaptation of PA (d) had less than 18 of age, (e) there was not a comparison between pre-and post-intervention, and (f ) it was not an RCT. These comparator categories were predefined before study selection to reduce conceptual heterogeneity. This approach has been previously adopted in systematic reviews of exercise interventions, where participant and therapist blinding is inherently unfeasible.

### Data sources and search strategy

A systematic literature search was conducted in June 2024 using the following online databases: PubMed, Cochrane Central Register of Controlled Trials, Scopus, SPORTDiscus, and Web of Science. The search was developed by synonyms of treatment resistant and physical activity or exercise and was performed by the followings search strategies: In PubMed (“major depression” OR “depression disorder” OR “depressive disorder” OR “treatment resistant depression” OR “non-remitted major depressive” OR “poorly responsive depressive” OR “treatment-resistant patients” OR “treatment-resistant major depressive”) AND (“physical activity” OR exercise); in Cochrane, Web of Science and SPORTDiscus “major depression” or “depression disorder” or “depressive disorder” or “treatment resistant depression” or “nonremitted major depressive” or “poorly responsive depressive” or “treatment-resistant patients” or “treatment-resistant major depressive”) and (“physical activity” or exercise); and in Scopus “major depression” OR “depression disorder” OR “depressive disorder” OR “treatment resistant depression” OR “nonremitted major depressive” OR “poorly responsive depressive” OR “treatment-resistant patients” OR “treatment-resistant major depressive” AND ““physical AND activity” OR “exercise””.

### Data screening and extraction

After duplicates were removed, the articles were revised by two independent authors (S.M-M and J.E-O) based on the titles and abstracts generated by the search strategy. After this procedure, any article that remained questionable was analyzed more thoroughly by reading the full text. The chosen articles were read and assessed using eligibility criteria. In case of discrepancies, the authors would reach a consensus. The following articles were revised using Rayyan Intelligent Systematic Review Software, which was developed specifically to expedite the initial screening of abstracts and titles using a semi-automated process. Likewise, in the publications, the details of the investigation appear to show that screening was conducted according to eligibility criteria.

A data extraction form was designed and piloted before its use. Information was extracted on the method (i.e., design, participants, intervention, outcome measures) and on the results (i.e., sample size, incidence rates, and number of events). When information was not available in the published trials, the corresponding author was requested to provide details. The data extraction was carried out by S.M-M and J.E-O (Data extraction form-supplementary data).

### Outcome parameters

The more significant outcome for this analysis was the improvement of depression by PA or exercise in TRD and NRD.

### Quality appraisal

For assessing included scientific articles, the Physiotherapy Evidence Database (PEDro) scale ( de Morton, 2009) and Oxford’s Evidence Levels were used ( Howick *et al*., 2009). The PEDro scale rates RCT on a scale from 0 (low quality) to 11 (high quality) related to scientific rigor ( de Morton, 2009). Each measure is rated “yes” or “no”. Items related to blinding of participants and assessors were considered in the context of exercise-based interventions, where blinding is often not feasible. Therefore, these items were not used to penalize study quality scores, and results were interpreted accordingly. This approach is consistent with previous systematic reviews of exercise-related research. For this reason, the maximum score on the modified PEDro 8-point scale was 7 (the highest), since the first item is not included in the total score. The qualitative ratings were adjusted to those used in previous exercise-related systematic reviews as follows: 6–7 = “excellent”; 5 = “good”; 4 = “moderate”; and 0–3 = “poor”. Oxford’s Evidence levels range from 1 to 5, with 1a being systematic reviews of high-quality RCT, 1b individual RCT with a narrow confidence interval, 2a systematic review of cohort studies, 2b individual cohort study, 3a systematic review of a case-control study, 3b individual case-control study, 4 case-series, and 5 being expert opinions ( Howick *et al*., 2009). Two researchers (S.M-M and J.E-O) inspected the methodology of each study independently. Disagreements regarding the appraisal of methodological quality were resolved through discussion between the reviewers.

In addition to the aforementioned tools, the Cochrane Risk of Bias 2 (RoB 2) tool was used to assess the methodological quality of parallel-group randomized trials (Higgins *et al.*, 2011). Five domains were evaluated: bias arising from the randomization process (Domain 1), bias due to deviations from intended interventions (Domain 2), bias due to missing outcome data (Domain 3), bias in measurement of the outcome (Domain 4), and bias in the selection of the reported result (Domain 5). For each domain, a judgment of ‘low risk’, ‘some concerns’, or ‘high risk’ was assigned. The overall risk of bias for each study was determined following Cochrane guidelines, with each study rated as ‘high risk’ if at least one domain was rated as ‘high risk’. Two reviewers (S.M-M and J.E-O) independently assessed the included studies, and discrepancies were resolved by consensus. For substudies derived from the TREAD trial, the published protocol was consulted to verify the pre-specification of outcomes ( Trivedi *et al.*, 2006).

### Grading the certainty of evidence (GRADE)

The certainty of evidence for the primary outcome (improvement in depressive symptoms) was assessed using the Grading of Recommendations, Assessment, Development and Evaluations (GRADE) framework (Guyatt *et al.*, 2011) The GRADE approach rates the certainty of evidence across four levels: high, moderate, low, and very low. Certainty was downgraded based on five domains: 1) Risk of bias: limitations in study design or conduct (e.g., lack of blinding, inadequate allocation concealment); 2) Inconsistency: unexplained heterogeneity across studies; 3) Indirectness: differences between the populations, interventions, or outcomes of interest and those studied; 4) Imprecision: wide confidence intervals or small total sample size; 5) Publication bias: likelihood that negative or null results remain unpublished.

### Heterogeneity evaluation

Heterogeneity across studies was assessed in a structured manner across three dimensions: clinical (population, intervention, comparator, outcome), methodological (study design), and statistical (effect measure, effect size, and Heterogeneity index [I ^2^] when estimable). The results of this assessment are presented in Table 4 and were used to determine the feasibility of a meta-analysis and to justify the narrative synthesis of the findings.

## Searching results

### Selection of studies

The database searches identified 10777 references (see *Extended data*, ( Etxaniz-Oses *et al*., 2023)). 3189 from PubMed, 1219 in Cochrane, 5698 in Web of Science, 567 in Scopus, and 104 in SPORTDiscus. After eliminating duplicates (n = 3973), a total of 6804 articles were removed by title, abstract, study design, population, or type of intervention. Consequently, 20 articles were assessed for eligibility, and the assessment was done in full text. Finally, 11 articles were included in the systematic review. These were published between 2002 and 2017, showing the importance of PA in TRD and NRD Flow diagram, Supplementary data). Between 2018 and 2024, a total of 1,973 articles were identified, of which 1,239 were excluded due to study type, 463 due to the study population, and 271 due to the study design. No articles were included from that period.

### Principal results and characteristics of studies

Physical activity as a predictor of various factors of mental illness, as well as producing benefits in depression was the highlight of the articles ( Carta *et al*., 2008; Greer *et al*., 2014, 2016; Mather *et al*., 2002; Mota-Pereira *et al*., 2011; Rethorst *et al*., 2013a, 2013b, 2016; Toups *et al*., 2011, 2017; Trivedi *et al*., 2011).

A total of 11 studies met the inclusion criteria. These comprised three independent RCTs (( Mota-Pereira *et al.*, 2011), n = 33; ( Mather *et al.*, 2002), n = 86; ( Carta *et al.*, 2008), n = 30) and one principal RCT (( Trivedi *et al.*, 2011), n = 126) with eight associated sub-studies (( Greer *et al.*, 2014), n = 39; ( Greer *et al.*, 2016) n = 106; ( Rethorst *et al.*, 2013b), n = 73; ( Rethorst *et al.*, 2013a), n = 122; ( Toups *et al.*, 2011), n = 70; ( Toups *et al.*, 2017), n = 119; ( Rethorst *et al.*, 2016), n = 122) reporting secondary outcomes from the same patient cohort (total unique participants across TREAD-related studies: n = 126). Thus, the total number of unique participants across all studies was 292 (241 from independent RCTs + 126 from TREAD, with eigth sub-studies reporting overlapping data from the TREAD cohort). When counting each sub-study separately (including overlapping TREAD participants), the aggregate participant count across all 11 reports is 567.

The supplemental data and Table 1 included the main characteristics of the accepted studies in this systematic review. First, it is important to highlight that eight of the 11 selected articles were from the same research project in the TRD or NRD population (n=275). Additionally, we have added a table that specifies the different definitions used to characterize the populations analyzed in each included article (Definitions of populations in the studies – Supplementary data). Therefore, no variability was observed in the population and study design ( *i.e.,* two groups comparing different volumes of exercise doses) ( Greer *et al*., 2014, 2016; Rethorst *et al*., 2013a, 2013b, 2016; Toups *et al*., 2011, 2017; Trivedi *et al*., 2011). Nevertheless, the other three articles were not part of the same research project ( Carta *et al*., 2008; Mather *et al*., 2002; Mota-Pereira *et al*., 2011). Concerning the type of PA assessed, while two articles conducted a novelty concurrent exercise intervention ( *i.e.,* including endurance and resistance training in the same session) ( Carta *et al*., 2008; Mather *et al*., 2002), an endurance training approach was observed in the other articles’ intervention, such as only aerobic continuous exercise (treadmill or cycle-ergometer) ( Greer *et al*., 2014, 2016; Rethorst *et al*., 2013a, 2013b, 2016; Toups *et al*., 2011, 2017; Trivedi *et al*., 2011), or weight-bearing strengthening exercise ( Mota-Pereira *et al*., 2011). None of the control groups performed any particular exercise, but pharmacological treatment ( Carta *et al*., 2008) or health-educational talks ( Mather *et al*., 2002).

**Table 1.** Characteristics of included studies and results

Study (year)	Outcome measure	Group	Baseline (mean ± SD)	Post-intervention (mean ± SD)	Effect size ( *d* of Cohen)	*P*-value
( Mota-Pereira *et al.*, 2011)	HAMD17	Exercise	19.32 ± 1.69	12.48 ± 1.50	SMD:-4.05 (high)	*P* < 0.05
		Control (TAU only)	13.00 ± 1.42	13.60 ± 0.96	SMD:+0.42 (low)	
	BDI	Exercise	24.68 ± 2.07	18.21 ± 2.35	SMD:-3.43(high)	*P* < 0.05
		Control (TAU only)	17.83 ± 1.93	22.13 ± 1.70	SMD:+2.23 (high)	
	GAF	Exercise	53.84 ± 2.55	61.89 ± 2.50	SMD:+ 3.16 (high)	*P* < 0.05
		Control (TAU only)	66.50 ± 2.76	61.06 ± 2.70	SMD:-1.97 (High)	
( Mather *et al.*, 2002)	HRSD (≥30% reduction)	Exercise	NR	55% achieved response	OR = 2.51	*P* = 0.05
		Health education + TAU	NR	33% achieved response		
( Trivedi *et al.*, 2011)	IDS-C _30_ (remission)	High-dose (16 KKW) + SSRI	33.3 ± 7.1	28.3% remission	NNT = 7.8	*P* = 0.06
		Low-dose (4 KKW) + SSRI	34.7 ± 7.8	15.5% remission		
	HRSD _17_ (change)	High-dose (16 KKW) + SSRI	17.8 ± 3.8	Change: -6.8	-6.8	*P* < 0.0001
		Low-dose (4 KKW) + SSRI	18.1 ± 3.8	Change: -4.9	-4.9	*P* < 0.0001
( Greer *et al.*, 2014)	SWM (between errors 8-box)	High-dose (16 KKW) + SSRI	NR	Fewer errors	Not reported	*P* < 0.04
		Low-dose (4 KKW) + SSRI	NR	More errors	Not reported	*P* < 0.04
	IED (Pre-ED errors)	High-dose (16 KKW) + SSRI	NR	NS change	Not reported	NS
		Low-dose (4 KKW) + SSRI	NR	More errors	Not reported	*P* < 0.04
( Greer *et al.*, 2016)	SF-36 Mental Health	High-dose (16 KKW) + SSRI	50.7 ± 13.9	67.2 ± 18.5	SMD:+1.19 (high)	*P* < 0.001
		Low-dose (4 KKW) + SSRI	49.1 ± 15.9	62.4 ± 21.0	SMD:+0.84 (high)	*P* < 0.001
	SAS-SR	High-dose (16 KKW) + SSRI	2.5 ± 0.4	2.1 ± 0.4	SMD:-1.00 (high)	*P* < 0.001
		Low-dose (4 KKW) + SSRI	2.4 ± 0.4	2.1 ± 0.5	SMD:-0.75 (moderate)	*P* < 0.001
	Q-LES-Q (general activities)	High-dose (16 KKW) + SSRI	60.2 ± 10.2	70.3 ± 11.9	SMD:+0.99 (high)	*P* < 0.001
		Low-dose (4 KKW) + SSRI	60.7 ± 10.9	70.2 ± 13.5	SMD:+0.87 (high)	*P* < 0.001
( Carta *et al.*, 2008)	WHOQOL-Bref (Physical)	Exercise	10.7 ± 2.3	12.9 ± 1.8	SMD:+0.96 (High)	*P* < 0.03
		Control (TAU only)	10.5 ± 3.3	10.5 ± 3.2	SMD:0.00 (Null)	NS
	WHOQOL-Bref (Psychological)	Exercise	10.8 ± 1.3	11.1 ± 1.8	SMD:+0.23 (low)	NS
		Control (TAU only)	11.7 ± 1.6	12.0 ± 1.9	SMD:+0.19 (low)	NS
( Rethorst *et al.*, 2013b)	TNF-α (predictor)	All participants	Higher baseline TNF-α → greater decrease in IDS-C	-	Parameter: -0.6155	*P* = 0.0023
	IL-1β (change correlation)	All participants	Change in IL-1β correlated with change in HRSD _17_	-	r_s = 0.24	*P* < 0.05
( Rethorst *et al.*, 2013a)	Total Insomnia score	High-dose (16 KKW) + SSRI	3.9 ± 2.3	Decreased over time	-	*P* < 0.0001
		Low-dose (4 KKW) + SSRI	4.5 ± 1.8	Decreased over time	-	*P* < 0.0001
	Sleep Onset Insomnia	High-dose (16 KKW) + SSRI	1.4 ± 1.2	Decreased over time	-	*P* < 0.002
		Low-dose (4 KKW) + SSRI	1.6 ± 1.1	Decreased over time	-	*P* < 0.002
	Hypersomnia	High-dose (16 KKW) + SSRI	0.7 ± 0.9	No significant change	-	NS
		Low-dose (4 KKW) + SSRI	0.7 ± 0.9	No significant change	-	NS
( Toups *et al.*, 2011)	BDNF (ng/ml)	High-dose (16 KKW) + SSRI	19.7 ± 5.4	19.3 ± 5.9	SMD: -0.07 (Null)	NS
		Low-dose (4 KKW) + SSRI	18.6 ± 6.5	19.2 ± 5.7	SMD: +0.09 (Null)	NS
	BDNF as predictor	All participants	Higher baseline BDNF → greater decrease in IDS-C	-	-	*P* = 0.0046
( Toups *et al.*, 2017)	MEI (Motivation)	High-dose (16 KKW) + SSRI	34.4 ± 12.5	51.5 ± 20.5	SMD: +1.37 (high)	*P*< 0.001
		Low-dose (4 KKW) + SSRI	34.9 ± 16.0	50.0 ± 16.1	SMD: +0.94 (high)	*P* < 0.001
	SHAPS (Anhedonia)	High-dose (16 KKW) + SSRI	2.8 ± 3.1	1.6 ± 2.9	SMD: -0.39 (low)	*P* = 0.013
		Low-dose (4 KKW) + SSRI	3.4 ± 3.0	2.2 ± 2.8	SMD: -0.40 (low)	*P* = 0.017
( Rethorst *et al.*, 2016)	IDS-C (exit LS mean)	Atypical depression (n=35)	38.3 ± 6.9	21.4 (LS mean)	MD: -16.9	*P* = 0.0735
		Non-atypical (n=87)	32.3 ± 7.0	24.5 (LS mean)	MD: -7.8	(interaction)

Abbreviations: BDI, Beck Depression Inventory; BDNF, Brain derived Neurotrophic Factor; CANTAB, Cambridge Neuropsychological Test Automated Battery; CI, Confidence Interval; d, Cohen's d effect size; GAF, Global Assessment of Functioning; HAMD _17_, 17-item Hamilton Depression Rating Scale; HRSD _17_, 17-item Hamilton Rating Scale for Depression; IDS-C _30_, 30-item Inventory of Depressive Symptomatology, Clinician-Rated; IED, Intra-dimensional/Extra-dimensional Shift; IFN-γ, Interferon-gamma; IL-1β, Interleukin-1 beta; IL-6, Interleukin-6; KKW, Kilocalories per kilogram of body weight per week; LS mean, Least squares mean; MEI, Motivation and Energy Inventory; NNT, Number Needed to Treat; NR, Not Reported; NS, Not Significant; OR, Odds Ratio; Q-LES-Q, Quality of Life Enjoyment and Satisfaction Questionnaire; RCT, Randomized Controlled Trial; SAS-SR, Social Adjustment Scale – Self-Report; SD, Standard Deviation; SF-36, 36-item Short-Form Health Survey; SHAPS, Snaith-Hamilton Pleasure Scale; SSRI, Selective Serotonin Reuptake Inhibitor; SMD, Standardized Mean Difference; SWM, Spatial Working Memory; TAU, Treatment As Usual; TNF-α, Tumor Necrosis Factor-alpha; TREAD, Treatment with Exercise Augmentation for Depression study; WHOQOL-Bref, World Health Organization Quality of Life – Brief version; η ^2^, Eta-squared.

In terms of the weekly frequency and time (volume) of PA, variability was shown in the studied articles (see *Extended data*, ( Etxaniz-Oses *et al*., 2023)). First, there was only one intervention, five days per week ( Mota-Pereira *et al*., 2011), whereas the rest of the articles were performed two days per week. Second, the duration of the session ranges from 45 to 60 minutes. In addition, the period of PA or exercise intervention to analyze the changes ranged from 10 ( Mather *et al*., 2002) to 12 weeks ( Greer *et al*., 2014, 2016; Mota-Pereira *et al*., 2011; Rethorst *et al*., 2013a, 2013b, 2016; Toups *et al*., 2011, 2017; Trivedi *et al*., 2011); but on the other hand, there was one article with a period of eight months of assessment ( Carta *et al*., 2008), and only one study presented a follow-up assessment after 24 weeks ( Mather *et al*., 2002). All the reviewed studies used moderate intensities for exercise and continuous endurance training controlled by a heart rate monitor ( Carta *et al*., 2008; Greer *et al*., 2014, 2016; Mota-Pereira *et al*., 2011; Rethorst *et al*., 2013a, 2013b, 2016; Toups *et al*., 2011, 2017) and rate of perceived exertion ( Mota-Pereira *et al*., 2011). However, in two studies, the intensity was not mentioned ( Carta *et al*., 2008; Mather *et al*., 2002), and another survey reported endurance training on a treadmill with an absolute exercise intensity of 5 km/h ( Mota-Pereira *et al*., 2011). In this sense, the rest of the articles performed two different exercise doses ( *i.e.,* low dose: 4 kcal/kg/week (KKW) equivalent to 75 min/week, and high dose: 16 KKW equivalent to 210 min/week ( Greer *et al*., 2014, 2016; Rethorst *et al*., 2013a, 2013b, 2016; Toups *et al*., 2011, 2017; Trivedi *et al*., 2011).

Overall, after PA intervention, in all but one of the reviewed articles ( Rethorst *et al*., 2016), depression symptoms and functioning parameters improved, in both TRD and NRD. The Hamilton Rating Scale for Depression (HRSD), Inventory of Depressive Symptoms-Clinical rated (IDS-C), Clinical Global Impression (CGI), and Geriatric Depression Scale (GDS), were the most widely used scales for depression screening ( Carta *et al*., 2008; Mather *et al*., 2002; Mota-Pereira *et al*., 2011; Rethorst *et al*., 2013a, 2013b). In addition, quality of life, as assessed with instruments such as the SF-36 and the World Health Organization Quality of Life (WHOQOL) also improved significantly ( Greer *et al*., 2016). Further, sleep quality ( Rethorst *et al*., 2013a), pro-inflammatory levels ( Rethorst *et al*., 2013b), and some biomarkers of depression ( Toups *et al*., 2011) showed better values after PA intervention. In addition, executive function and working memory improved with increasing exercise volume. Still, psychomotor speed, attention, visual memory, spatial planning, instrumental and expressive roles, and cognitive function improved irrespective of the type or dose of exercise participants undertook ( Greer *et al*., 2014, 2016). The remission of the participants also improved and was achieved in response to antidepressants with the PA as an adjuvant program on the different traits of TRD and NRD. Thus, in one of the RCTs, it was shown that 29.5% of the participants achieved remission ( Trivedi *et al*., 2011), and the remission was analyzed with different scales, such as HRSD, IDS-C, CGI, and GDS ( Mather *et al*., 2002; Mota-Pereira *et al*., 2011).

Rethorst *et al.* (2016) examined whether the atypical depression subtype predicted exercise response in 122 participants from the TREAD study. The authors did not find a significant improvement in depressive symptoms associated with the atypical subtype (small effect, p = 0.0735). However, a non-significant trend toward a greater response was observed in the atypical depression group assigned to the high-dose exercise condition. The authors concluded that atypical depression could be a potential moderator of exercise response, but that these findings should be considered hypothesis-generating due to limited statistical power.

Adherence to exercise interventions and dropout rates varied across studies. In the three independent RCTs, Mather *et al.* (2002) reported the highest adherence to supervised group exercise (67% mean attendance) and no dropouts. Mota-Pereira *et al.* (2011) reported a high adherence rate (91%) defined as completing ≥50% of prescribed walks per week, with a 6% dropout rate. In the TREAD study ( Trivedi *et al.*, 2011), adherence was dose-dependent: the low-dose group (4 KKW) had a median adherence of 99.4%, while the high-dose group (16 KKW) had a median adherence of 63.8%. The overall dropout rate during the 12-week acute phase was 17.2% (21/122), with main reasons including inconvenience/time constraints (n = 10), non-compliance (n = 7), and desire for alternative treatment (n = 5). In TREAD substudies, attrition was higher when additional procedures were required (e.g., blood draw for biomarkers: 42-44% dropout; cognitive testing: 20% dropout from eligible participants). ( Carta *et al.*, 2008) did not report adherence or dropout rates. These findings highlight that adherence to high-dose exercise is challenging in TRD/NRD populations, and that dropout rates are substantial, particularly in studies requiring additional assessments.

A meta-analysis was not conducted because several studies did not report the statistical data required for quantitative synthesis (e.g., standard deviations or effect estimates for remission/response). For this reason, a meta-analysis can actually assess heterogeneity, which can be taken into consideration in the final quality of evidence and interpretation.

### Risk of bias

Table 2 summarizes the PEDro scale and Oxford’s Evidence levels for the included articles in this systematic review. All the articles except one ( Carta *et al*., 2008), were classified as excellent. Inadequate concealed allocation to groups and the lack of similarity in the comparison groups at baseline were the methodological limitations in the article, which was considered a “good” quality description. All papers were RCT, and for this reason, Oxford’s Evidence levels were 1b in six articles ( Greer *et al.*, 2016; Mather *et al.*, 2002; Rethorst *et al.*, 2013a, 2016; Toups *et al.*, 2017; Trivedi *et al.*, 2011) and five 2b ( Carta *et al.*, 2008; Greer *et al.*, 2014; Mota-Pereira *et al.*, 2011; Rethorst *et al.*, 2013b; Toups *et al.*, 2011). All studies performed comparisons of both intra-group (pre- and post-PA-intervention) and inter-group (exercise vs. control group) when presented. Further, inclusion and exclusion criteria were defined, and blinding eligibility was presented.

**Table 2.** Assessment of the PEDro scale and Oxford’s Evidence levels of the included scientific articles that analyzed TRD and NRD after PA intervention.

PEDro ratings										Oxford’s Evidence Levels
	1	2	3	4	5	6	7	8	Total	
References										
( Mota-Pereira *et al.*, 2011)	Y	Y	Y	Y	Y	Y	Y	Y	7	2b
( Mather *et al.*, 2002)	Y	Y	Y	Y	Y	Y	Y	Y	7	1b
( Trivedi *et al.*, 2011)	Y	Y	Y	Y	Y	Y	Y	Y	7	1b
( Greer *et al.*, 2014)	Y	Y	Y	Y	Y	Y	Y	Y	7	1b
( Greer *et al.*, 2016)	Y	Y	Y	Y	Y	Y	Y	Y	7	1b
( Carta *et al.*, 2008)	Y	Y	N	N	Y	Y	Y	Y	5	2b
( Rethorst *et al.*, 2016)	Y	Y	Y	Y	Y	Y	Y	Y	7	1b
( Rethorst *et al.*, 2013a)	Y	Y	Y	Y	Y	Y	Y	Y	7	1b
( Rethorst *et al.*, 2013b)	Y	Y	Y	Y	Y	Y	Y	Y	7	2b
( Toups *et al.*, 2011)	Y	Y	Y	Y	Y	Y	Y	Y	7	2b
( Toups *et al.*, 2017)	Y	Y	Y	Y	Y	Y	Y	Y	7	1b

Abbreviations: N, no; NRD, non-remitted depression; PA, physical activity: TRD, treatment resistant depression; Y, yes.

(1) Eligibility criteria were specified.

(2) Participants were randomized groups.

(3) Allocation was concealed.

(4) The groups were similar at baseline regarding the most important prognostic indicators.

(5) Measures of at least one key outcome were obtained from more than 85% of participants initially allocated to groups.

(6) All participants from whom outcome measures were available received the treatment or control condition as allocated or, where this was not the case, data for at least one key outcome was analyzed by “intention to treat”.

(7) The results of between-group statistical comparisons are reported for at least one key outcome.

(8) The study provides both point measures and measures of variability for at least one key outcome.

Considering the RoB 2 tool across the 11 included studies, five main trials ( Mather *et al.*, 2002; Mota-Pereira *et al.*, 2011; Rethorst *et al.*, 2013a, 2016; Trivedi *et al.*, 2011) presented an overall risk of bias rated as ‘some concerns’, primarily due to lack of participant blinding (unavoidable in exercise interventions) and non-pre-specified post-hoc analyses. The remaining six studies were rated as having ‘high risk’ of overall bias (Risk of Bias RoB2, Supplementary data).

### Summary of findings

The quality of the evidence varied according to the outcome assessed. For the primary outcome of depressive symptoms, the quality was moderate ( Mather *et al.*, 2002; Mota-Pereira *et al.*, 2011; Trivedi *et al.*, 2011), based on three clinical trials with moderate risk of bias. For the secondary outcomes, the quality was low for sleep quality ( Rethorst *et al.*, 2013a) and very low for psychosocial functioning, cognition, biomarkers, quality of life and positive valence symptoms, due to serious methodological limitations, inconsistency across studies, and indirectness of the measurements ( Carta *et al.*, 2008; Greer *et al.*, 2014, 2016; Rethorst *et al.*, 2013b, 2016; Toups *et al.*, 2011, 2017) ( Table 3).

**Table 3.** Summary findings (SoF), GRADE quality of evidence by outcome.

Outcome	Number of studies (Participants)	GRADE quality	Justification
Depressive symptoms	3 (251)	Moderate	RCTs, moderate risk of bias; consistent results
Sleep quality	1 (122)	Low	RCT; moderate risk of bias; self-reported outcome
Quality of life	1 (30)	Very low	RCT; high risk of bias; small sample (n = 30); self-report
Psychosocial functioning	1 (106)	Very low	High risk of bias (self-report, no blinding)
Cognition	1 (39)	Very low	High risk of bias (small subsample, completers only)
Biomarkers	2 (143)	Very low	High risk of bias (42-44% attrition, completers only); inconsistency
Positive valence/Predictors	2(241)	Very low	High risk of bias; inconsistency; imprecision

Abbreviations: GRADE, Grading of Recommendations, Assessment, Development and Evaluation; RCT, Randomized Control Trial.

### Heterogenity

As shown in Table 4, clinical, methodological, and statistical heterogeneity were assessed across the three studies reporting depressive symptom outcomes. Marked heterogeneity was observed across populations (i.e., adults with TRD vs NRD), interventions (i.e., individual walking vs group-based exercise vs dose-comparison designs), and outcome measures (i.e., HAMD17, HRSD, and IDS-C _30_). Statistical heterogeneity between the studies by Mather *et al.* (2002), and Mota-Pereira *et al.* (2011) was low (I ^2^ = 0%). However, the ( Greer *et al.*, 2014, 2016; Rethorst *et al.*, 2013b, *et al.*, 2013a, 2016; Toups *et al*., 2011, 2017; Trivedi *et al.*, 2011) studies could not be included in this pooled estimate due to the use of a different effect measure. Furthermore, this study was not independent of the other TREAD-derived sub-analyses included in this review. Consequently, we opted for a narrative synthesis rather than a meta-analysis. On the other hand, the study by Carta *et al.* (2008) presented the following characteristics: Clinically, it included women aged 40-60 years with TRD, a group-based exercise intervention (2 sessions/week of 60 minutes for 32 weeks) versus TAU, and the outcome was quality of life (WHOQOL-Bref physical domain). Methodologically, it was a parallel-group RCT (2:1 allocation ratio) with unreported randomization, no allocation concealment, no intention-to-treat (ITT) analysis, and no reporting of losses to follow-up. Statistically, the effect measure was the mean difference, showing an improvement of +2.2 points in the exercise group (p < 0.03). As this was a single study, I ^2^ could not be calculated.

**Table 4.** Heterogeneity of the studies

Domain	( Mota-Pereira *et al.*, 2011)	( Mather *et al.*, 2002)	( Trivedi *et al.*, 2011) and substudies	Heterogeneity judgment
Clinical heterogeneity				
Population	Treatment-resistant Depression (≥9-15 months without remission)	Treatment-resistant depression (53-91 years)	Non-remitted MDD after adequate SSRI trial (18-70 years)	High
Intervention	Walking 30-45 min, 5 days/week (1 supervised)	Group exercise, 2 sessions/week, 45 min	High-dose (16 KKW) vs low-dose (4 KKW) aerobic exercise	High
Outcome measure	HAMD17 (change from baseline)	HRSD (response ≥30% reduction)	IDS-C _30_ (remission ≤12)	High
Methodological heterogeneity				
Design	Parallel RCT (2 arms)	Parallel RCT (2 arms)	Parallel dose-comparison RCT (2 arms)	Moderate
Statistical heterogeneity				
Effect measure (original)	Mean difference	Odds Ratio (OR)	Number Needed to Treat (NNT)	High
Effect size (95% CI)	-7.44 ( *P*< 0.05)	2.51 (1.00-6.38)	7.8 (p = 0.06)	Moderate
I ^2^	-	-	-	0% (low heterogeneity between Mota-Pereira and Mather)

Abbreviations: CI, Confidence Interval; HAMD _17_, 17-item Hamilton Depression Rating Scale; HRSD, Hamilton Rating Scale for Depression; IDS-C _30_, 30-item Inventory of Depressive Symptomatology, Clinician-Rated; I ^2^, Heterogeneity index; KKW, Kilocalories per kilogram of body weight per week; MDD, Major Depressive Disorder; NNT, Number Needed to Treat; OR, Odds Ratio; RCT, Randomized Controlled Trial.

## Discussion

The objective of this study was to investigate the effects of PA intervention in TRD and NRD, describing the different parameters such as psychological, physical, and mental function and quality of life ( Greer *et al*., 2014, 2016; Mather *et al*., 2002; Mota-Pereira *et al*., 2011; Rethorst *et al*., 2013a, 2013b, 2016; Toups *et al*., 2011, 2017; Trivedi *et al*., 2011). The review determined 11 articles and identified that 1) PA intervention as an adjuvant program could improve different traits of TRD and NRD, 2) improvements in depressive symptoms were reported in 10 of 11 included studies, but the independent evidence base is very limited: eight studies derived from the same TREAD project, and only three independent studies exist. Secondary outcomes such as quality of life, sleep quality, and executive function were reported in single studies. A GRADE assessment indicated very low certainty evidence. Therefore, no strong conclusion about remission or secondary benefits can be drawn from the current evidence and 3) according to the FITT principle, there was some variability in the PA intervention, and except for one article ( Carta *et al*., 2008), they all were classified as excellent in terms of quality description.

A previous meta-analysis has found an interesting association between PA and incident depression, even below the public health recommendations, with an additional benefit when minimum recommendations were met ( Pearce *et al*., 2022). Thus, in the present systematic review, even though different types of intervention ( *i.e.,* concurrent or endurance training) were performed in the reviewed articles, the depression decreased via HRSD ( Mather *et al*., 2002; Mota-Pereira *et al*., 2011), and the quality of life increased ( Carta *et al*., 2008; Greer *et al*., 2016), irrespective of PA type. Concerning only resistance intervention in patients with depression, previous studies have compared aerobic *vs.* nonaerobic exercise training and observed that both groups reduced depression scores, but with no significant difference between groups ( Doyne *et al*., 1987; Martinsen *et al*., 1989). Taking into account all those above and the World Health Organization PA guidelines for people with chronic conditions ( Bull *et al*., 2020), these results suggest to include both resistance and endurance training in the adjuvant PA programs for TRD and NRD population, as it might result in better outcomes as seen in other populations ( Silva *et al*., 2015; Stanton & Reaburn, 2013). In this sense, the prefrontal cortex, anterior cingulate cortex, hippocampus, and corpus callosum emerge as possible neural markers that benefit from exercise in people with depression ( Gujral *et al*., 2017). These findings support the idea that during exercise, skeletal muscle releases valuable molecules that activate signals from skeletal muscle to different tissues, including the brain, highlighting that inactivity and loss of muscle mass may render the brain vulnerable to neurological dysfunction and disease ( Delezie & Handschin, 2018; Isaac *et al*., 2021). Regarding the frequency of weekly sessions, interventions ranged from two to five sessions, with the most frequently used being 2.6 sessions per week. This information was not observed in other systematic reviews, which reported that three sessions had the highest weekly frequency ( Perraton *et al.*, 2010; Stanton & Happell, 2013). Our results could be associated with TRD and NRD patients’ poorer physical condition and chronicity over time compared to those patients with a diagnosis of only depression. The duration of the interventions in the articles was between 10 and 12 weeks in almost all of them ( Greer *et al*., 2014, 2016; Mather *et al*., 2002; Mota-Pereira *et al*., 2011; Rethorst *et al*., 2013a, 2013b, 2016; Toups *et al*., 2011, 2017; Trivedi *et al*., 2011). However, another article used an extended eight-month intervention ( Carta *et al*., 2008). Other research on patients with depression used interventions for more than eight weeks ( Perraton e *t al.*, 2010). Yet, it may be that an intervention with a duration of 10-12 weeks would be sufficient to improve depression in TRD and NRD.

A limitation of the included studies is the lack of long-term follow-up in most. Of the 11 studies analyzed, only ( Mather *et al.*, 2002) included a follow-up assessment (at 24 weeks), and did not report complete quantitative data on the evolution of depressive symptoms at that time point. None of the other studies (including the TREAD substudies) included follow-up beyond the intervention period (10-32 weeks). Given the chronic and recurrent nature of TRD, this is a critical gap that future research must address. It remains unknown whether the benefits of exercise are maintained over the long term, whether continued exercise is required to sustain the effect, or whether a rebound effect occurs upon cessation of the intervention as has been observed in other populations (Firth *et al.*, 2018).

Regarding the intensity of exercise, in the current study, all the reviewed articles performed moderate-intensity training interventions ( Carta *et al*., 2008; Greer *et al*., 2014, 2016; Mather *et al*., 2002; Mota-Pereira *et al*., 2011; Rethorst *et al*., 2013a, 2013b, 2016; Toups *et al*., 2011, 2017; Trivedi *et al*., 2011), with no studies including high-intensity training. Thus, recent investigations promote the relevance of exercise intensity and modality ( *i.e.,* high-intensity interval training) to induce a higher metabolic myokine effect on brain health ( Calverley *et al*., 2020; Hashimoto *et al*., 2021).

Three articles carried out a comparison between the control (no exercise intervention) and experimental group ( Carta *et al*., 2008; Mather *et al*., 2002; Mota-Pereira *et al*., 2011); the rest of the articles used a comparison with different doses of exercise ( Greer *et al*., 2014, 2016; Rethorst *et al*., 2013a, 2013b, 2016; Toups *et al*., 2011, 2017; Trivedi *et al*., 2011). In the articles that compared the control and experimental groups, the experimental groups observed better results than the control groups. In the same way, the groups that performed a high dose of exercise improved more than those that performed a low dose.

The present systematic review of RCTs analyzing the effects of PA intervention in patients with TRD and NRD determined the benefits of PA not only reducing depressive symptoms but also in other variables related to quality of life, such as sleep quality, executive and cognitive function, and memory. In summary, based on moderate-quality evidence from three independent RCTs, structured exercise programs may be considered as an adjunctive intervention for depressive symptoms in patients with TRD and NRD, particularly within the context of individualized care and shared decision-making. However, clinicians should be aware that the evidence base is limited: one of the three studies ( Mather *et al.*, 2002), focused on older adults with poorly responsive depression rather than strictly defined TRD, and evidence for secondary outcomes (quality of life, sleep, cognition, biomarkers) is of low or very low quality, derived from single studies or non-independent TREAD sub-analyses. Therefore, this remains a preliminary suggestion rather than a strong clinical recommendation. Further high-quality, adequately powered RCTs specifically targeting TRD and NRD populations are urgently needed to confirm these findings, establish optimal PA protocols, and compare exercise with other established augmentation strategies before stronger recommendations can be made.

## Limitations and strengths

The most important limitation are: 1) Eight of the 11 studies are from the same project. No attempt was made to retrieve data that could be used for a meta-analysis because the magnitude of the missing data was large. 2) The clinical and methodological heterogeneity observed across the studies ( Table 4) is an important limitation of this review. Differences in populations, interventions, and outcomes preclude direct comparison of the results and justify the narrative synthesis. Even so, it is essential to include them because they show different variables that may help to identify TRD or NRD. 3) Absence of adverse event reporting. None of the 11 included studies systematically reported data on adverse events related to the exercise intervention, such as musculoskeletal events (injuries, joint pain, stress fractures), cardiovascular events (arrhythmias, syncope, ischemic events), and adverse psychological events (exercise-induced anxiety, frustration from failing to meet goals, worsening of mood, or excessive fatigue).

However, this is the first review to examine these two populations regarding exercise, and we have observed a lack of interventions in both.

## Data Availability

All data underlying the results are available as part of the article and no additional source data are required. Zenodo: Physical activity in the treatment-resistant depression and non-remitted depression: a systematic review of randomized controlled trials, https://doi.org/10.5281/zenodo.10044119 ( Etxaniz-Oses *et al*., 2023). This project contains the following extended data:
- Extended data-supplementary data. Main characteristic of studies that analyzed resistant treatment or non-remitted depression and physical activity • Data extraction form-supplementary data. Method used to extract data from publications - Flow diagram-supplementary data. Flowchart of the systematic review following the rules of PRISMA • Prisma_2020_abstract_checklist. • Definitions of populations in the studies – Supplementary data Zenodo: PRISMA checklist for ‘Physical activity in the treatment-resistant depression and non-remitted depression: a systematic review of randomized controlled trials’, https://doi.org/10.5281/zenodo.10044119 ( Etxaniz-Oses *et al*., 2023). Data are available under the terms of the Creative Commons Zero “No rights reserved” data waiver (CC0 1.0 Public domain dedication).
