# Supplementary material for: Physical activity in the treatment-resistant depression and non-remitted depression: a systematic review of randomized controlled trials
Source: F1000Res. 2026 Jul 1;12:1517. Originally published 2023 Nov 27. [Version 8] doi: 10.12688/f1000research.140970.8 (PMC13550888; doi:10.12688/f1000research.140970.8)
Supplement: Peer review history

# Reviewer report: Reviewer response for version 8

**DOI:** 10.5256/f1000research.200392.r497994

**Published:** 2026-07-06

**Authors:** Gerasimos N. Konstantinou

**Affiliations:** 1 Centre of Addiction and Mental Health, Toronto, Canada

The manuscript has substantially improved. No additional comments.

Are the rationale for, and objectives of, the Systematic Review clearly stated?

Partly

Is the statistical analysis and its interpretation appropriate?

Partly

If this is a Living Systematic Review, is the ‘living’ method appropriate and is the search schedule clearly defined and justified? (‘Living Systematic Review’ or a variation of this term should be included in the title.)

Not applicable

Are sufficient details of the methods and analysis provided to allow replication by others?

Partly

Are the conclusions drawn adequately supported by the results presented in the review?

Partly

Reviewer Expertise:

Interventional Psychiatry, Depression, Clinical Psychiatry, Brain Stimulation

I confirm that I have read this submission and believe that I have an appropriate level of expertise to confirm that it is of an acceptable scientific standard.

# Reviewer report: Reviewer response for version 7

**DOI:** 10.5256/f1000research.195170.r472669

**Published:** 2026-04-10

**Authors:** Gerasimos N. Konstantinou

**Affiliations:** 1 Centre of Addiction and Mental Health, Toronto, Canada

Thank you for inviting me to review this systematic review that investigates the effects of physical activity (PA) and exercise as adjuvant interventions, supplementing ongoing pharmacological treatment, in adults with treatment-resistant depression (TRD) and non-remitted depression (NRD).

 The review is registered on PROSPERO (CRD42022298347) and follows the PRISMA 2020 reporting framework. A search of five electronic databases (PubMed, Cochrane Central Register of Controlled Trials, Scopus, SPORTDiscus, and Web of Science) conducted in June 2024 identified 10,777 references, from which 11 randomized controlled trials (RCTs) were ultimately included. Methodological quality was appraised using a modified PEDro scale (items 1–8, excluding blinding items) and Oxford Evidence Levels. A meta-analysis was not performed, with the authors citing insufficient statistical data across studies and substantial heterogeneity.

 The 11 included RCTs were published between 2002 and 2017. A critical observation is that eight of the eleven studies (Greer et al. 2014, 2016; Rethorst et al. 2013a, 2013b, 2016; Toups et al. 2011, 2017; Trivedi et al. 2011) originate from the same research project, the TREAD (TReatment with Exercise Augmentation for Depression) trial. The three independent studies (Carta et al. 2008; Mather et al. 2002; Mota-Pereira et al. 2011) were conducted in distinct populations using varied protocols. The main finding is that PA intervention, primarily moderate-intensity aerobic or concurrent training delivered 2–5 times per week over 10–12 weeks, was associated with improvements in depressive symptoms in 10 of 11 studies, alongside secondary gains in quality of life, sleep quality, executive function, and pro-inflammatory biomarkers.

 Please see below my comments.

 1) The review combines two clinically and operationally distinct populations, TRD (inadequate response to at least two adequate antidepressant trials) and NRD (partial response, defined here as a Hamilton Depression Rating Scale [HDRS] score ≥14 at screening), under a single synthesis without adequately justifying this decision. The distinction between these populations is not trivial: TRD implies pharmacological failure after multiple adequate trials, whereas NRD as operationalized in the TREAD studies reflects partial response. These populations may have different pathophysiological profiles and could respond differently to exercise augmentation. The authors should explicitly justify the rationale for pooling these two groups, ideally citing evidence that the mechanisms of exercise benefit are expected to be equivalent across TRD and NRD. Alternatively, results should be presented separately for TRD and NRD where possible.

 2) The authors acknowledge that "the definitions of TRD and NRD are unclear" and cite Harbi (2012), a narrative review, as the primary source for the TRD criterion of ≥2 failed antidepressant trials. However, substantial literature now exists on TRD staging and consensus definitions, including Canadian expert consensus frameworks and the GSRD staging model. The review should anchor its operational TRD definition to the most current consensus criteria and explicitly document how each included study's population was classified as TRD versus NRD, rather than relying on how primary authors self-labeled their populations.

 3) The introduction does not adequately explain why PA might be effective specifically in TRD/NRD as opposed to treatment-responsive MDD. The neurobiological mechanisms discussed (myokine release, prefrontal cortex/hippocampus neuroplasticity) are referenced in the discussion but not the introduction. Given that TRD/NRD may involve distinct neuroinflammatory profiles and reduced neuroplasticity, a brief mechanistic argument in the introduction for why adjuvant PA is plausible in this group would substantially strengthen the rationale.

 4) The manuscript does not describe the data extraction instrument used to capture study characteristics, outcomes, or FITT (Frequency, Intensity, Time, Type) parameters from included studies. It is unclear whether extraction was standardized across reviewers, what items were extracted, or how disagreements in extraction were handled. A description of the data extraction process and the variables captured (or provision of the extraction template as supplementary material) is required for replication.

 5) Although the search was updated in June 2024, the 11 included studies all date from 2002–2017. This seven-year gap between the most recent included study and the search date warrants comment. Were there eligible studies published between 2017 and 2024 that met inclusion criteria but were not retrieved? The authors should document whether any studies from 2018–2024 were excluded and, if so, provide the reason for exclusion (ideally in the PRISMA flow diagram).

 6) The quality appraisal relies on the PEDro scale and Oxford Evidence Levels. Reviewer Schmitter recommended the Cochrane Risk of Bias 2 (RoB 2) tool, which is now the standard in Cochrane-aligned systematic reviews. While the PEDro scale has demonstrated validity for exercise RCTs and agreement between PEDro and the Cochrane RoB tool is only moderate, the PEDro scale does not evaluate selective outcome reporting, other bias domains, or provide a domain-specific risk-of-bias profile. The authors' justification for not adopting RoB 2, that "PEDro and Oxford's evidence levels are quality appraisals the journal accepts", is procedurally adequate but methodologically insufficient. Authors should at minimum acknowledge this as a limitation.

 7) No GRADE (Grading of Recommendations, Assessment, Development, and Evaluations) assessment was performed. For a systematic review making clinical recommendations about an adjunct treatment strategy in a vulnerable population, absence of a GRADE framework means the strength of evidence supporting conclusions cannot be evaluated. This is a significant omission. Authors should apply GRADE to the primary outcome (depressive symptom improvement) and report the resulting certainty of evidence rating (e.g., low, very low), which would likely reflect the limited independent evidence base.

 8) Despite not performing a formal meta-analysis, a minimum standard for systematic reviews is the systematic presentation of extracted quantitative data from included studies. The manuscript delegates the "main characteristics of studies" entirely to supplementary data on Zenodo. Readers cannot assess the evidence without access to a structured evidence table, including sample sizes, mean baseline and post-intervention depression scores (with standard deviations where available), and primary outcome results, directly in the main manuscript. This table should be moved from supplementary material to the main text, as recommended by Reviewer Dinas.

 9) While heterogeneity across all 11 studies precludes a single meta-analysis, the eight TREAD-derived studies used highly standardized protocols (same population, same exercise modality, same outcome measures: HDRS, IDS-C) with two dose conditions (4 kcal/kg/week vs. 16 kcal/kg/week). A subset meta-analysis comparing these two dose conditions within TREAD, and/or a separate meta-analysis of the three independent studies, would have been methodologically feasible and informative. The authors should explicitly acknowledge why such subset analyses were not attempted, or alternatively, attempt them.

 10) Even in a narrative synthesis, it is expected that heterogeneity across included studies be described in a structured manner. No I² or other heterogeneity index is reported or estimated. The authors should at minimum provide a descriptive heterogeneity table (clinical: population, intervention, outcome; methodological: design; statistical: where estimable) to justify the narrative-only approach.

 11) No assessment of publication bias was performed (e.g., funnel plot, Egger's test, or qualitative assessment using Begg's rank correlation). Given that all 11 included studies reported positive or neutral results (with only Rethorst et al. 2016 finding no significant improvement), this is an important omission. Publication bias is a known issue in exercise-depression research, and the absence of any assessment, even qualitative, should be explicitly acknowledged as a limitation.

 12) Individual study effect sizes (Cohen's d, Hedges' g) are not reported for any included study, nor are confidence intervals. The Noetel et al. (2024) network meta-analysis found moderate effect sizes (Hedges' g ranging from −0.42 to −0.62) for various exercise modalities in general depression populations. A meaningful comparison with the TRD/NRD-specific evidence requires extracting or calculating effect sizes from available primary study data. Authors should provide effect sizes where calculable from reported data.

 15) The abstract states: "This review highlights the potential of PA intervention as an adjuvant program to improve various traits in TRD and NRD. The remission of depression seems to be higher after PA intervention, showing improvements in quality of life, sleep quality, executive function, and vitality." This conclusion is misleading for the following reasons:

- TREAD dominance: 8 of 11 studies are from the same research project with the same patient population (n=275). The effective independent evidence base for the conclusion that PA improves depressive outcomes in TRD/NRD consists of only three unique studies. A conclusion framed as if 11 independent trials have confirmed this association is scientifically inaccurate.
- Secondary outcomes from single studies: Quality of life improvement is documented in one study (Greer et al. 2016), sleep quality in one study (Rethorst et al. 2013a), and pro-inflammatory biomarkers in one study (Rethorst et al. 2013b). Listing these as general findings in the abstract creates an impression of convergent evidence that does not exist.
- Remission: The only remission rate reported is 29.5% from Trivedi et al. 2011, a single TREAD study. This cannot support a general conclusion about remission.

Authors must revise the abstract conclusions to accurately reflect the limited independent evidence, clearly caveat outcomes to the number of studies reporting them, and distinguish TREAD-derived findings from independent replications.

 16) The discussion concludes: "Clinicians should consider structured exercise programs as part of multidisciplinary management in TRD and NRD." Given that the independent evidence base consists of three studies, one of which (Mather et al. 2002) examined "older adults with poorly responsive depressive disorder" rather than clinically defined TRD, this recommendation exceeds the evidentiary support. Without GRADE rating, the strength of this recommendation is unquantifiable. The authors should downgrade this statement to a preliminary suggestion pending further evidence, using appropriate hedging language (e.g., "may be considered in the context of individualized care" rather than "should").

 17) Rethorst et al. (2016), the one study reporting no significant improvement in depressive symptoms after PA, is referenced but not substantively discussed. This study's negative finding is clinically relevant and warrants dedicated discussion, including consideration of whether the population (atypical depressive features as a moderator) provides insights into who may or may not benefit from exercise augmentation in TRD/NRD.

 18) Only one included study (Mather et al. 2002) includes a follow-up assessment (at 24 weeks). The long-term durability of PA-induced improvements in TRD/NRD is entirely unaddressed in the conclusions. Given the chronic and relapsing nature of TRD, this is a clinically critical gap that must be prominently acknowledged.

 19) No data on adverse events (e.g., musculoskeletal injury, cardiovascular events, exercise-related psychological distress) were extracted or reported across any included study. For a clinical recommendation regarding an active intervention in a psychiatrically vulnerable population, adverse event data are essential. Authors should report adverse event data where available in included studies, and explicitly note if such data were not reported in primary studies as a limitation.

 20) The paper operates on the premise that TRD and NRD are related but distinct conditions worthy of joint examination. However, the operational definitions used across included studies are inconsistent. The TREAD studies define their population as NRD using the criterion of HDRS ≥14 after an adequate antidepressant trial, which is qualitatively different from TRD requiring failure of ≥2 antidepressant classes. The authors should produce a supplementary table clarifying how each included study operationally defined its population, which criteria for TRD or NRD were applied, and how the authors classified each study as TRD, NRD, or both.

 21) The search was performed in June 2024, yet all 11 included studies were published between 2002 and 2017. This seven-year evidence gap is unexplained. Given the expansion of exercise-psychiatry research over the past decade, it is implausible that no eligible RCTs were published between 2017 and 2024. The authors should provide a documented accounting of studies from this period that were identified but excluded, along with reasons for exclusion.

 22) Earlier reviewer versions flagged a major discrepancy: an independent Scopus search using the provided algorithm yielded 72,153 publications, whereas the authors reported only 567. The authors corrected the Scopus search string in a subsequent version, yielding 567. However, the current manuscript does not clearly document the corrected Scopus search string or provide evidence of this correction in the main text. Authors should include the corrected Scopus search string in the methods and verify its reproducibility.

 23) The total number of participants across all included studies is not reported in the main text. This is a basic descriptive requirement for systematic reviews. Authors should report the aggregate participant count (also noting that a substantial proportion, n=275, derives from the TREAD project).

 24) Exercise adherence and dropout rates, both clinically critical parameters for translating PA findings to TRD/NRD care, are not systematically reported. In a population with treatment resistance, adherence barriers may be particularly relevant. Authors should extract and tabulate adherence/dropout data where available.

Are the rationale for, and objectives of, the Systematic Review clearly stated?

Partly

Is the statistical analysis and its interpretation appropriate?

Partly

If this is a Living Systematic Review, is the ‘living’ method appropriate and is the search schedule clearly defined and justified? (‘Living Systematic Review’ or a variation of this term should be included in the title.)

Not applicable

Are sufficient details of the methods and analysis provided to allow replication by others?

Partly

Are the conclusions drawn adequately supported by the results presented in the review?

Partly

Reviewer Expertise:

Mood Disorders, Interventional Psychiatry, Psychopathology, Psychoimmunology

I confirm that I have read this submission and believe that I have an appropriate level of expertise to state that I do not consider it to be of an acceptable scientific standard, for reasons outlined above.

# Reviewer report: Reviewer response for version 6

**DOI:** 10.5256/f1000research.194515.r446545

**Published:** 2026-01-09

**Authors:** Petros C Dinas

**Affiliations:** 1 FAME Laboratory, School of Physical Education, Sport Science and Dietetics, University of Thessaly, Thessaly, Greece

Regarding my comment referring to not conducting a meta-analysis, I suggest the following revision: "A meta-analysis was not conducted because the several included studies did not report the statistical data required for quantitative synthesis (e.g., standard deviations or effect estimates for remission/response)." This is because a meta-analysis can actually assess heterogeneity, which can be taken into consideration in the final quality of evidence and interpretation. I also suggest the authors to include a limitation as following: "No attempt was made to retrieve data that could be used for a meta-analysis, due to the missing data magnitude was large."

Are the rationale for, and objectives of, the Systematic Review clearly stated?

Yes

Is the statistical analysis and its interpretation appropriate?

Not applicable

If this is a Living Systematic Review, is the ‘living’ method appropriate and is the search schedule clearly defined and justified? (‘Living Systematic Review’ or a variation of this term should be included in the title.)

Not applicable

Are sufficient details of the methods and analysis provided to allow replication by others?

Partly

Are the conclusions drawn adequately supported by the results presented in the review?

Partly

Reviewer Expertise:

Systematic review and meta-analysis

I confirm that I have read this submission and believe that I have an appropriate level of expertise to confirm that it is of an acceptable scientific standard.

# Response

**Published:** 2026-01-11

**Authors:** SARA MALDONADO-MARTIN

**Affiliations:** PHYSICAL EDUCATION AND SPORT, UNIVERSITY OF THE BASQUE COUNTRY (UPV/EHU), VITORIA-GASTEIZ, BASQUE COUNTRY, Spain

Dear reviewer, thank you very much for your "approved" statement and commentary. We will include your suggestions in the next version of the manuscript once the editor permits us.

# Reviewer report: Reviewer response for version 4

**DOI:** 10.5256/f1000research.172539.r332807

**Published:** 2024-10-23

**Authors:** Miguel Jacinto

**Affiliations:** 1 ESECS-Polytechnic of Leiria, Leiria, Portugal

Thank you very much for the opportunity to review the manuscript ‘Physical activity in the treatment-resistant depression and non-remitted depression: a systematic review of randomized controlled trials. The article is written in a simple and clear manner and may be suitable for indexing in the F1000Research. I'll leave some comments that I think are pertinent to improving the manuscript and clarifying some fundamental points so that it can be indexed:

 The number of studies that were extracted from the search should appear in the results section of the abstract.

 The keywords should be different to the words used in the title.

 Disorder is different from disability.

 What hypotheses are being studied?

 The introduction does not make it clear to the author what gap in the literature the authors aim to fill.

 What does this study add to the literature?

 Did the authors strictly follow the protocol submitted to PROSPERO?

 Were studies with sports interventions excluded?

 What is the difference between the two types of studies included in the review? Why were only these two types of studies included?

 The eligibility criteria should be written in more detail.

 The start and end date of the literature search is unclear.

 It is not clear when the studies searched were conducted.

 What is the reason for choosing these databases?

 Why is the search different in the databases?

 The reason for removing items from the PEDro scale is unclear.

 The authors could proceed to carry out a meta-analysis

 What tool was used to assess the risk of bias?

Are the rationale for, and objectives of, the Systematic Review clearly stated?

No

Is the statistical analysis and its interpretation appropriate?

Partly

If this is a Living Systematic Review, is the ‘living’ method appropriate and is the search schedule clearly defined and justified? (‘Living Systematic Review’ or a variation of this term should be included in the title.)

Partly

Are sufficient details of the methods and analysis provided to allow replication by others?

No

Are the conclusions drawn adequately supported by the results presented in the review?

No

Reviewer Expertise:

systematic-reviews

I confirm that I have read this submission and believe that I have an appropriate level of expertise to confirm that it is of an acceptable scientific standard, however I have significant reservations, as outlined above.

# Response

**Published:** 2024-10-31

**Authors:** SARA MALDONADO-MARTIN

**Affiliations:** PHYSICAL EDUCATION AND SPORT, UNIVERSITY OF THE BASQUE COUNTRY (UPV/EHU), VITORIA-GASTEIZ, BASQUE COUNTRY, Spain

ANSWERS TO REVIEWER- **Miguel Jacinto** **-1**

 Physical activity in the treatment-resistance depression and non-remitted depression: a systematic review of randomized controlled trials

 We express our sincere gratitude for taking the time to review the manuscript and for the invaluable suggestions provided to enhance it. Following your recommendations, we have been able to improve the manuscript and have implemented the following changes:

 1.The number of studies that were extracted from the search should appear in the results section of the abstract. Dear reviewer, the number of studies has been presented in the methods section. As you suggested, we will change it and present it in the results section.

 2. The keywords should be different to the words used in the title.

 Amended. Repeated keywords have been replaced

 3. Disorder is different from disability.

 Dear reviewer. Agree with you in your assertion. However, we want to say that the major depressive disorder may lead to disability. In the present disorder, people with major depression may suffer a functional disadvantage affected by that condition. To clarify, we have changed the second part of the sentence- Major depressive disorder (MDD) is one of the most prevalent mental illnesses in the world, which may lead to disability.

 4. What hypotheses are being studied?

 Dear reviewer, as you know, a hypothesis is not strictly necessary in a systematic review as in an experimental study. However, it can be helpful. Therefore, as you suggested, we have added a hypothesis at the end of the introduction.

 5. The introduction does not make it clear to the author what gap in the literature the authors aim to fill.

 Dear reviewer, to date, there are no systematic reviews showing results according to the effects of physical activity and exercise on people with Treatment-resistant depression. There are some studies but no systematic review. We think that it is important to show that exercise is also a valuable tool in this population.

 6. What does this study add to the literature?

 Dear reviewer, this study aims to examine the effects of exercise interventions in people with TRD and NRD. This population is relatively small, and as noted in the review, interventions involving exercise are limited. This review offers important contributions to the existing literature and concludes that exercise may improve the condition. However, further research in this area is necessary to strengthen the evidence.

 7. Did the authors strictly follow the protocol submitted to PROSPERO?

 Yes, the systematic review is registered as CRD42022298347 and was accepted for implementation by Prospero, and we followed the submitted protocol.

 8. Were studies with sports interventions excluded?

 Thank you for your comment. As explained in the section on PICOS questions, we searched for and included any intervention involving physical activity or exercise. However, interventions involving sports were not considered or included in the search strategy. To clarify, we have added a sentence related to this issue.

 9. What is the difference between the two types of studies included in the review?

 Randomized trials may sometimes omit the control group and conduct multiple groups with different types of treatment as opposed to RCTs, which are studies in which there is a control group.

 10. Why were only these two types of studies included?

 To include randomized clinical studies in a systematic review is important for several reasons:

- Minimization of bias: Randomization helps reduce selection and confounding bias, allowing the intervention and control groups to be comparable at the start of the study.
- Greater internal validity: Randomized studies generally offer greater internal validity, meaning that the results are more reliable and can be attributed with greater certainty to the evaluated intervention.
- More robust evidence: Including randomized trials provides a solid basis for making clinical recommendations, as these studies are considered the gold standard for evaluating the effectiveness of an intervention.
- Consistency in quality: By focusing on randomized controlled trials, consistency in the methodological quality of the included studies is ensured, which facilitates comparison of results.
- Better generalizability: Although generalizability may be limited, well-designed RCTs tend to have a more representative sample of the general population, allowing better extrapolation of findings.

For these reasons, systematic reviewers prefer to focus on randomized studies to obtain more robust and reliable conclusions about the efficacy of an intervention.

 11. The eligibility criteria should be written in more detail.

 Dear reviewer, we have used the PICOS strategy, and all the components have been specifically explained. If you think that something is missing, we would appreciate more information.

 12.The start and end date of the literature search are unclear.

 Thank you for your comment. In the Data Sources and Search Strategy section, we provided information regarding your question: A systematic literature search was conducted in June 2024. During that month, the search and selection of articles were carried out.

 13. It is not clear when the studies searched were conducted.

 We have answered this question in the previous one.

 14. What is the reason for choosing these databases?

 We consider these databases among the most widely utilized in our scientific field, and we believe that by incorporating five reputable databases, we would successfully meet the objectives of this systematic review.

 15. Why is the search different in the databases?

 In each database, the search strategy has been adjusted because Boolean operators such as AND/OR function differently across the platforms.

 16. The reason for removing items from the PEDro scale is unclear.

 Dear reviewer, removing items 5 and 7 from the PEDro scale in a systematic review might be justified for several reasons:

- **Item 5 (Blinding of Participants)**: If blinding participants is deemed impractical or irrelevant to the specific intervention being studied, it could be argued that this item does not significantly impact the study's findings.
- **Item 7 (Blinding of Assessors)**: Similar to item 5, if the nature of the intervention or outcome measurement does not allow for blinding of assessors (e.g., subjective outcomes) or if it has been shown that assessors’ bias does not significantly affect results, this item may be considered less critical.

Removing these items might be based on a rationale that the overall integrity of the reviewed studies remains intact despite the absence of these specific quality indicators.

 17. The authors could proceed to carry out a meta-analysis.

 Dear reviewer, thank you for your comment. However, according to the chosen articles, our data are insufficient to perform the statistical procedure required for meta-analysis. Further, the diversity of the primary outcomes makes it even more difficult to combine data derived from the different studies. An explanation regarding this issue is presented in the results.

 18. What tool was used to assess the risk of bias?

 Dear reviewer, we have used the PEDro scale to appraise the quality of evidence in clinical trials. Higher scores indicate a lower risk of bias and more reliable findings. It is true that while this scale addresses important methodological aspects, it does not cover all potential sources of bias.

# Reviewer report: Reviewer response for version 3

**DOI:** 10.5256/f1000research.170568.r317444

**Published:** 2024-09-19

**Authors:** Petros C Dinas

**Affiliations:** 1 FAME Laboratory, School of Physical Education, Sport Science and Dietetics, University of Thessaly, Thessaly, Greece

1. Regarding my first comment, the PICOS in the comparator section, is still confusing.

 2. The title of the first section of the results did not change.

 3. The justification of not performing a meta-analysis is still insufficient. Also, the new addition of “what interests us” is confusing. The methodology of a systematic review and a meta-analysis focus on removing bias of the process rather than add bias.

Are the rationale for, and objectives of, the Systematic Review clearly stated?

Yes

Is the statistical analysis and its interpretation appropriate?

Not applicable

If this is a Living Systematic Review, is the ‘living’ method appropriate and is the search schedule clearly defined and justified? (‘Living Systematic Review’ or a variation of this term should be included in the title.)

Not applicable

Are sufficient details of the methods and analysis provided to allow replication by others?

Partly

Are the conclusions drawn adequately supported by the results presented in the review?

Partly

Reviewer Expertise:

Systematic review and meta-analysis in biological studies

I confirm that I have read this submission and believe that I have an appropriate level of expertise to confirm that it is of an acceptable scientific standard, however I have significant reservations, as outlined above.

# Response

**Published:** 2024-09-24

**Authors:** SARA MALDONADO-MARTIN

**Affiliations:** PHYSICAL EDUCATION AND SPORT, UNIVERSITY OF THE BASQUE COUNTRY (UPV/EHU), VITORIA-GASTEIZ, BASQUE COUNTRY, Spain

ANSWERS TO REVIEWERS

 Petros C Dinas-2

 Physical activity in the treatment-resistance depression and non-remitted depression: a systematic review of randomized controlled trials

 We greatly appreciate the reviewer's time and insight. Your comments and our subsequent revision have improved our manuscript. We have reviewed the entire manuscript and made changes to improve it.

 1.    Regarding my first comment, the PICOS in the comparator section is still confusing.

**Author Response:**

 Dear reviewer, in your previous review, you mentioned- "While the authors state that only randomized trials are accepted as a study design, the comparator section in PICOS includes pre-post intervention comparisons. This can not actually be considered as a comparator in an RCT design. I realize that it may be used for calculating Δ scores if this is the case, but I suggest removing it to avoid confusion."

 As you suggested, we removed the pre vs. post-comparison from this version.

 2.    The title of the first section of the results did not change.

**Author Response:**

 As you have suggested the main title of the results section now is called "Searching results."

 3.    The justification of not performing a meta-analysis is still insufficient. Also, the new addition of "what interests us" is confusing. The methodology of a systematic review and a meta-analysis focus on removing bias of the process rather than add bias.

**Author Response:**

 We have changed the paragraph, adding a final sentence, and deleted what seems confusing: Although we understand that the meta-analysis of the results of the different studies may provide objective global information on specific variables, according to the chosen articles, our data are insufficient to perform the statistical procedure required for meta-analysis. Further, the diversity of the primary outcomes makes it even more difficult to combine data derived from the different studies. In this sense, the final decision was influenced by heterogeneity among studies, as well as studies' quality or the risk of bias

# Response

**Published:** 2025-12-28

**Authors:** SARA MALDONADO-MARTIN

**Affiliations:** PHYSICAL EDUCATION AND SPORT, UNIVERSITY OF THE BASQUE COUNTRY (UPV/EHU), VITORIA-GASTEIZ, BASQUE COUNTRY, Spain

We are pleased to submit Version 6 of our manuscript titled: "Physical activity in treatment-resistant depression and non-remitted depression: a systematic review of randomized controlled trials".

**Opening statement:**

 In this revised version (Version 6), we have carefully reviewed and thoroughly addressed all comments raised by both reviewers in Versions 3 and 4. All previously discussed partial points have now been explicitly revised, clarified, and incorporated into the manuscript. Each comment has been responded to in full below, with precise indications of where changes were made in the revised text.

 We sincerely thank the reviewers for their constructive and detailed feedback, which has significantly improved the clarity, methodological transparency, and overall quality of our manuscript.

**Response to Reviewers' Comments**

 Reviewer: Petros C. Dinas

Comment 1 – PICOS comparator still confusing

 We thank the reviewer for insisting on further clarification of the PICOS framework. We fully agree that clarity in the comparator definition is essential.

 In the revised manuscript, we have explicitly defined the comparator conditions as follows:

1.  Treatment as Usual: Participants continued their standard pharmacological treatment and usual clinical care without any structured physical activity component.
2. Active Control: Participants received an alternative intervention unrelated to exercise (e.g., health education, relaxation therapy, or low-intensity psychological support) to control attention, time, and social interaction effects.
3. No-Intervention/Wait-list Control: Participants did not receive any additional structured intervention, although baseline pharmacological treatment continued.

These categories were pre-specified before study selection to reduce conceptual heterogeneity. The clarification has been incorporated in the Methods section (Study eligibility criteria, page 4).

Comment 2 – Title of first Results section

 We agree with the reviewer. The first subsection of the Results is now titled "Searching results", followed by clearly defined subsections:

- Selection of studies
- Principal results and characteristics of studies
- Risk of bias

This structure now aligns with PRISMA recommendations and improves readability.

Comment 3 – Justification for not conducting a meta-analysis

 We thank the reviewer for this   critical comment. We have revised and simplified the justification for not conducting a meta-analysis to improve clarity.

 Although a meta-analysis could theoretically be attempted, we concluded that it would not be methodologically appropriate due to:

- Substantial clinical, methodological, and outcome heterogeneity across studies
- Insufficient statistical data required for quantitative synthesis (e.g., missing standard deviations or effect estimates for remission/response)

This justification is now clearly stated in the Results section (page 7), and all subjective or potentially confusing wording has been removed.

 Reviewer: Miguel Jacinto

Comment 1 – Disorder vs Disability

 We agree with the reviewer. To avoid conceptual confusion, we have revised the wording to clarify that while major depressive disorder is a psychiatric condition, it may lead to significant functional impairment and disability. The sentence has been rephrased accordingly (Introduction).

Comment 2 – PEDro scale interpretation

 We thank the reviewer for this comment. Items related to blinding of participants and assessors were discussed in the context of exercise-based interventions, where blinding is often not feasible. Rather than excluding their relevance, we have clarified this methodological limitation and interpreted PEDro scores accordingly in the risk-of-bias assessment section (page 10). In this sense, it presents concerns about scoring without penalizing studies for inherent limitations in exercise trials.

**Summary of Changes:**

- PICOS comparator categories clarified and explicitly described.
- Results section reorganized and subsection titles aligned with PRISMA guidelines.
- Justification for not performing a meta-analysis is simplified and clarified.
- The wording is refined to differentiate between disorder and disability.
- The PEDro scale methodology and interpretation were fully clarified to address concerns regarding blinding.
- Minor editorial, stylistic, and grammatical adjustments were made throughout to ensure consistency and clarity.

We believe that Version 6 fully addresses all reviewer concerns and represents a precise, rigorous, and methodologically transparent manuscript.

 We sincerely appreciate the reviewers' time and constructive feedback, which have significantly improved the quality and clarity of our work. We hope that the revised manuscript is now suitable for publication in F1000Research.

 We appreciate your consideration.

# Reviewer report: Reviewer response for version 2

**DOI:** 10.5256/f1000research.168194.r307276

**Published:** 2024-08-02

**Authors:** Michele Schmitter

**Affiliations:** 1 Depression Expertise Center, Pro Persona Mental Health Care, Nijmegen, The Netherlands

The authors performed a systematic review to study the effectiveness of exercise or physical activity interventions as an adjunct to pharmacotherapy in treatment-resistant and non-remitted depression. Although the topic is of interest to the scientific community, the article has several major shortcomings that need to be addressed before Indexing can be considered.

**General Remarks**

 Please clarify ambiguous statements (e.g., page 3, line 3; page 4, outcome parameter), and use clear and concise language (e.g., page 3, line 6).

**Introduction**

 The relevance of exercise or physical activity as an add-on therapy for NRD or TRD is not well developed. The authors should explain why exercise could be a good add-on treatment for NRD and TRD. They could use the available evidence showing that exercise is effective for MDD (e.g., Noetel et al., 2024; Morres et al., 2019) to argue that it might also be effective for NRD and TRD, but no systematic review has been conducted so far. Additionally, it would be useful to define physical activity and exercise, and elaborate on the different exercise protocols and how they relate to the current evidence. Finally, the study by Krogh et al. (2017) did not include patients with TRD and NRD.

**Methods**

 Intervention: Please elaborate on the types of interventions included in this review. What kinds of interventions were included? Supervised exercise treatment, activity tracker or online modules, exercise advice, single exercise sessions, etc.?

 Comparator: What kinds of control conditions were included?

 Outcome: Other outcomes that the authors discuss later should be mentioned here.

 Study Design: Include within-person designs. However, it is unclear why the authors chose within-person designs if they only included RCTs.

 Quality Appraisal: The quality appraisal is not appropriate. I recommend using other quality assessment tools (e.g., Cochrane Risk of Bias Tool).

 Selection of Studies: A table summarizing the selected studies should be included in the manuscript, not in the supplements.

**Results**

 Characteristics of Studies: The provided citations are confusing. The authors state that eight of the studies are from the same project but cite all 11 studies afterward. Only those studies from the same project should be cited here. The same applies to the rest of this paragraph. The authors state that two studies (i.e., Carta and Mather) used concurrent exercise interventions and the others used “traditional” interventions, again citing Carta and Mather. Moreover, "concurrent" and "traditional" interventions are not appropriate terms in this context.

 Characteristics of Exercise Interventions: “The period of assessment,” where the authors refer to the assessment of depressive symptoms, should be stated elsewhere. Instead, the intervention period should be mentioned here.

 Meta-Analysis Statement: The statement explaining why the authors do not perform a meta-analysis is misplaced in the methods section. Please avoid using informal language.

**Discussion**

 Since eight of the 11 studies used the same population, there are actually only three studies showing that exercise can be an effective add-on treatment to reduce depressive symptoms in TRD and NRD. The amount of evidence is therefore very limited, which the authors should discuss. A more hesitant optimism towards exercise as an add-on treatment for TRD and NRD would be more appropriate considering the limited evidence so far.

Are the rationale for, and objectives of, the Systematic Review clearly stated?

No

Is the statistical analysis and its interpretation appropriate?

Not applicable

If this is a Living Systematic Review, is the ‘living’ method appropriate and is the search schedule clearly defined and justified? (‘Living Systematic Review’ or a variation of this term should be included in the title.)

Not applicable

Are sufficient details of the methods and analysis provided to allow replication by others?

No

Are the conclusions drawn adequately supported by the results presented in the review?

No

Reviewer Expertise:

Exercise, depression, RCTs

I confirm that I have read this submission and believe that I have an appropriate level of expertise to state that I do not consider it to be of an acceptable scientific standard, for reasons outlined above.

# Response

**Published:** 2024-08-18

**Authors:** SARA MALDONADO-MARTIN

**Affiliations:** PHYSICAL EDUCATION AND SPORT, UNIVERSITY OF THE BASQUE COUNTRY (UPV/EHU), VITORIA-GASTEIZ, BASQUE COUNTRY, Spain

**ANSWER TO REVIEWER**

**August, 2024**

**Michele Schmitter**, Depression Expertise Center, Pro Persona Mental Health Care, Nijmegen, The Netherlands 

**Reviewer Comment:**

 The authors performed a systematic review to study the effectiveness of exercise or physical activity interventions as an adjunct to pharmacotherapy in treatment-resistant and non-remitted depression. Although the topic is of interest to the scientific community, the article has several major shortcomings that need to be addressed before Indexing can be considered.

**General Remarks**. Please clarify ambiguous statements (e.g., page 3, line 3; page 4, outcome parameter), and use clear and concise language (e.g., page 3, line 6).

- **Author Response: **
- Dear reviewer, considering all your requests, we have clarified the matter throughout the manuscript. Thank you very much for your review. Your comments have been constructive in improving the manuscript.

**Reviewer Comment:**

**Introduction**

- The relevance of exercise or physical activity as an add-on therapy for NRD or TRD is not well developed. The authors should explain why exercise could be a good add-on treatment for NRD and TRD. They could use the available evidence showing that exercise is effective for MDD (e.g., Noetel et al., 2024; Morres et al., 2019) to argue that it might also be effective for NRD and TRD, but no systematic review has been conducted so far.
  - **Author Response: **
  - Dear reviewer, thank you very much for your suggestions. We have added the recent systematic review and meta-analysis by Noetel et al. 2024 to the article's introduction.
  - The discussion explains the cause-effect of exercise in the brain and people with MDD or anyone.

**Reviewer Comment:**

- Additionally, it would be useful to define physical activity and exercise.
  - **Author Response: **
  - Definitions for physical activity and exercise have been amended in the introduction.
-  Elaborate on the different exercise protocols and how they relate to the current evidence.
  - **Author Response: **
  - Amended. It has been added that effective treatments (different kinds of exercise) are proportional to the intensity prescribed.
- Finally, the study by Krogh et al. (2017) did not include patients with TRD and NRD.

(Krogh et al.2017).

-
  - **Author Response:**
  - Dear reviewer, thank you for your message; we have deleted this reference from the introduction and changed the corresponding paragraph.

**Reviewer Comment:**

**Methods**

- Intervention: Please elaborate on the types of interventions included in this review. What kinds of interventions were included? Supervised exercise treatment, activity tracker or online modules, exercise advice, single exercise sessions, etc.?
  - **Author Response:**
  - Thank you for your message. We have considered explaining more: " **I**ntervention **:** any type of PA or exercise intervention that includes supervised exercise, online sessions, unsupervised activities, or recommendations."
- Comparator: What kinds of control conditions were included?
  - **Author Response:**
  - Thank you for your message. We have considered your proposal and written down the characteristics of the control participants.
- Outcome: Other outcomes that the authors discuss later should be mentioned here.
  - **Author Response:**
  - Thanks for your message; we have added some complementary information.
- Study Design: Include within-person designs. However, it is unclear why the authors chose within-person designs if they only included RCTs.
  - **Author Response:**
  - Dear reviewer, this information is not already in the manuscript.
- Quality Appraisal: The quality appraisal is not appropriate. I recommend using other quality assessment tools (e.g., Cochrane Risk of Bias Tool).
  - **Author Response:**
  - Dear reviewer, thank you for your recommendation. However, PEDro and Oxford's evidence levels are quality appraisals the journal accepts. If we change with your recommendation, we should also change all the written results related to this issue. Anyway, we will take into account your recommendation for the following work.
- Selection of Studies: A table summarizing the selected studies should be included in the manuscript, not the supplements.
  - **Author Response:**
  - Thank you for the suggestion. We will ask the journal's editor whether this suggestion is possible.

**Reviewer Comment:**

**Results**

- Characteristics of Studies: The provided citations are confusing. The authors state that eight of the studies are from the same project but cite all 11 studies afterward. Only those studies from the same project should be cited here.
  - **Author Response:**
  - Dear reviewer. Thank you very much for your suggestion; we have changed the citations to only mention the eight regarding the same project, not the 11 ones.
- The same applies to the rest of this paragraph. The authors state that two studies (i.e., Carta and Mather) used concurrent exercise interventions, and the others used "traditional" interventions, again citing Carta and Mather. Moreover, "concurrent" and "traditional" interventions are inappropriate in this context.
  - **Author Response:**
  - Thank you for your message; we have appropriately changed the citations.
- Characteristics of Exercise Interventions: "The period of assessment," where the authors refer to the assessment of depressive symptoms, should be stated elsewhere. Instead, the intervention period should be mentioned here.
  - **Author Response:**
  - Thank you for your message. We wanted to emphasize the exercise intervention's duration, not the evaluation period's duration. To clarify this term, we have changed the "period of assessment" to the "period of physical activity or exercise intervention."
- Meta-Analysis Statement: The statement explaining why the authors do not perform a meta-analysis is misplaced in the methods section. Please avoid using informal language.
  - **Author Response:**
  - Dear reviewer. According to another reviewer's suggestion, we have also changed this paragraph in the following way: "Although we understand that the meta-analysis of the different studies' results may provide objective global information on specific variables, according to the chosen articles, the data we have are insufficient to perform the statistical procedure required for meta-analysis. Further, the diversity of the primary outcomes makes it even more difficult to combine data derived from the different studies. In this sense, a systematic review allows us to include those variables related to "what interests us" that cannot be meta-analyzed due to insufficient cohorts or test statistics."

**Reviewer Comment:**

**Discussion**. Since eight of the 11 studies used the same population, there are actually only three studies showing that exercise can be an effective add-on treatment to reduce depressive symptoms in TRD and NRD. Therefore, the amount of evidence is very limited, which the authors should discuss. A more hesitant optimism towards exercise as an add-on treatment for TRD and NRD would be more appropriate considering the limited evidence so far.

- **Author Response:**
- Dear reviewer, we have changed the last paragraph of the discussion, adding what you recommend regarding the limited evidence. In this sense, we have also changed the conclusion in the abstract and manuscript.

# Reviewer report: Reviewer response for version 2

**DOI:** 10.5256/f1000research.168194.r306169

**Published:** 2024-07-25

**Authors:** Petros C Dinas

**Affiliations:** 1 FAME Laboratory, School of Physical Education, Sport Science and Dietetics, University of Thessaly, Thessaly, Greece

1. While the authors state that only randomized trials are accepted as a study design, the comparator section in PICOS, includes pre-post intervention comparisons. This can not actually be considered as a comparator in a RCT design. I realize that it may be used for calculating Δ scores, if this is the case, but I suggest removing it, to avoid confusion.

 2. The title of the first section of the results, should also include searching results.

 3. The justification provided by the authors regarding "not performing a meta-analysis" is not clear. What does mean "a global view of the scientific evidence"? Reasons of not performing a meta-analysis usually include lack of available evidence or large heterogeneity to reply the research question. If the authors are able to retrieve numerical data from the included studies, a meta-analysis would provide more accurate estimations of the effect, than a narrative data synthesis.

Are the rationale for, and objectives of, the Systematic Review clearly stated?

Yes

Is the statistical analysis and its interpretation appropriate?

Not applicable

If this is a Living Systematic Review, is the ‘living’ method appropriate and is the search schedule clearly defined and justified? (‘Living Systematic Review’ or a variation of this term should be included in the title.)

Not applicable

Are sufficient details of the methods and analysis provided to allow replication by others?

Partly

Are the conclusions drawn adequately supported by the results presented in the review?

Partly

Reviewer Expertise:

Systematic review and meta-analysis in biological studies

I confirm that I have read this submission and believe that I have an appropriate level of expertise to confirm that it is of an acceptable scientific standard, however I have significant reservations, as outlined above.

# Response

**Published:** 2024-07-27

**Authors:** SARA MALDONADO-MARTIN

**Affiliations:** PHYSICAL EDUCATION AND SPORT, UNIVERSITY OF THE BASQUE COUNTRY (UPV/EHU), VITORIA-GASTEIZ, BASQUE COUNTRY, Spain

We greatly appreciate the reviewer's time and insight. Your comments and our subsequent revision have improved our manuscript. We have reviewed the entire manuscript and made changes to improve it.

 1.    While the authors state that only randomized trials are accepted as a study design, the comparator section in PICOS includes pre-post intervention comparisons. This can not actually be considered as a comparator in a RCT design. I realize that it may be used for calculating Δ scores, if this is the case, but I suggest removing it, to avoid confusion.

 •    Amended. Agree with you

 2.    The title of the first section of the results, should also include searching results.

 •    Amended

 3.     The justification provided by the authors regarding "not performing a meta-analysis" is not clear. What does mean "a global view of the scientific evidence"? Reasons for not performing a meta-analysis usually include lack of available evidence or large heterogeneity to reply the research question. If the authors are able to retrieve numerical data from the included studies, a meta-analysis would provide more accurate estimations of the effect, than a narrative data synthesis.

 •    Dear reviewer, thank you for your comment. However, according to the chosen articles, the data we have are insufficient to perform the statistical procedure required for meta-analysis. Further, the diversity of the primary outcomes makes it even more difficult to combine data derived from the different studies. We have changed the corresponding sentence to clarify more.

# Reviewer report: Reviewer response for version 1

**DOI:** 10.5256/f1000research.154379.r278378

**Published:** 2024-06-03

**Authors:** Petros C Dinas

**Affiliations:** 1 FAME Laboratory, School of Physical Education, Sport Science and Dietetics, University of Thessaly, Thessaly, Greece

1. The sections of "Eligibility Criteria" and "Identification and selection of studies" should be merged with the PICOS approach, or a section of the selection method should be created. As it is stated now both sections contain eligibility criteria.

 2. It's been more than two years from the searching date. This requires an update. A searching on PubMed on the 3rd June 2024 yield 3188 publications, indicating that potential eligible papers may have been published the last 2 years.

 3. It is not clear whether the screening of the eligible publications was also based on abstract and full texts reading.

 4. In PRISMA flowchart, it is reported that studies that were not clinical trials were excluded. However, in the eligibility criteria is reported that experimental studies is an accepted type of publications. Please define if only clinical trials were included in the systematic review.

 5. For clarity, I suggest the results section to include sub-sections for searching, selection, and risk of bias results, main outcomes as well as main characteristics of the included studies.

 6. The Reviewer performed a searching in Scopus on the 3rd June 2024, using the given algorithm. This search yield 72153 publications. However, in the results section is reported that Scopus yield 0 results during the search. Please check if the searching in Scopus is correct.

 7. It is confusing what was the "comparator" in the included studies. The eligibility criteria are not clear in terms of this, which consequently provides an unclear view of the results in relation to the research question. In other words the conclusion of the study is based on mixed data of comparison between exercisers and non exercisers and between exercisers and exercisers as controls.

 8. Based on the main results provided in the supplement, the authors should consider a meta-analysis. If not, a justification of not using a meta-analysis should be given and the type of data synthesis should be reported.

 9. The Discussion does not provide any limitations of the systematic review

Are the rationale for, and objectives of, the Systematic Review clearly stated?

Yes

Is the statistical analysis and its interpretation appropriate?

Not applicable

If this is a Living Systematic Review, is the ‘living’ method appropriate and is the search schedule clearly defined and justified? (‘Living Systematic Review’ or a variation of this term should be included in the title.)

Not applicable

Are sufficient details of the methods and analysis provided to allow replication by others?

Partly

Are the conclusions drawn adequately supported by the results presented in the review?

Partly

Reviewer Expertise:

Systematic review and meta-analysis in biological studies

I confirm that I have read this submission and believe that I have an appropriate level of expertise to state that I do not consider it to be of an acceptable scientific standard, for reasons outlined above.

# Response

**Published:** 2024-06-04

**Authors:** SARA MALDONADO-MARTIN

**Affiliations:** PHYSICAL EDUCATION AND SPORT, UNIVERSITY OF THE BASQUE COUNTRY (UPV/EHU), VITORIA-GASTEIZ, BASQUE COUNTRY, Spain

Thank you so much for your comments. Hopefully we will have the opportunity to answer you to improve the paper.

# Response

**Published:** 2024-06-20

**Authors:** SARA MALDONADO-MARTIN

**Affiliations:** PHYSICAL EDUCATION AND SPORT, UNIVERSITY OF THE BASQUE COUNTRY (UPV/EHU), VITORIA-GASTEIZ, BASQUE COUNTRY, Spain

SYSTEMATIC REVIEW

 Physical activity in the treatment-resistance depression and non-remitted depression: a systematic review of randomized controlled trials

 We greatly appreciate the reviewer's time and insight. Your comments and our subsequent revision have improved our manuscript. We have reviewed the entire manuscript and made changes to improve it.

**Reviewer comment: **The "Eligibility Criteria" and "Identification and selection of studies" sections should be merged with the PICOS approach, or a section of the selection method should be created. As it is stated now, both sections contain eligibility criteria.

**Author response:** Amended, we have reorganized this section.

**Reviewer comment**: It's been more than two years since the search date. This requires an update. A search on PubMed on the 3rd of June 2024 yielded 3188 publications, indicating that potentially eligible papers may have been published in the last 2 years.

**Author response: **Dear reviewer. Thanks for your message. This paper was submitted one year ago (2023), and your review was performed just a few weeks ago. This point justifies the date of our search data. Agree with you we have conducted a new search data with the same search strategy and the data have been updated.

**Reviewer comment:** It is not clear whether the screening of the eligible publications was also based on abstract and full-text reading.

**Author response: **Amended. We have better explained this point in the Data Screening and Extraction.

**Reviewer comment**: In PRISMA flowchart, it is reported that studies that were not clinical trials were excluded. However, in the eligibility criteria, it is reported that experimental studies is an accepted type of publications. Please define if only clinical trials were included in the systematic review.

**Author response: **Many thanks for your comment. We have corrected the PRISMA flowchart.

**Reviewer comment:** For clarity, I suggest the results section to include sub-sections for searching, selection, and risk of bias results, main outcomes as well as main characteristics of the included studies.

**Author response: **Many thanks. Now is everything more straightforward.

**Reviewer comment**: The Reviewer performed a searching in Scopus on the 3rd June 2024, using the given algorithm. This search yield 72153 publications. However, in the results section is reported that Scopus yield 0 results during the search. Please check if the searching in Scopus is correct.

**Author response: **Dear reviewer, you can find below an image of the research. 567 document were found with SCOPUS data base. We have changed the search or Scopus to better adjust it: "major depression" OR "depression disorder" OR "depressive disorder" OR "treatment resistant depression" OR "nonremitted major depressive" OR "poorly responsive depressive" OR "treatment-resistant patients" OR "treatment-resistant major depressive" AND " physical AND activity " OR " exercise ".

**Reviewer comment:** It is confusing what was the "comparator" in the included studies. The eligibility criteria are not clear in terms of this, which consequently provides an unclear view of the results in relation to the research question. In other words the conclusion of the study is based on mixed data of comparison between exercisers and non exercisers and between exercisers and exercisers as controls.

**Author response: **Thank you for your comment. You are right. We compare intra groups and inter groups. In this sense, we have add more information. **C**omparator **: **intra-group (pre- vs. post-intervention) and inter-group (control vs. exercise intervention).

**Reviewer comment:** Based on the main results provided in the supplement, the authors should consider a meta-analysis. If not, a justification of not using a meta-analysis should be given and the type of data synthesis should be reported.

**Author response: **Thank you for your comment. In the methods have added a paragraph related to that. Although we understand that the meta-analysis of the results of the different studies may provide objective global information on certain variables, the present study aims not to perform a meta-analysis but to provide a global view of the scientific evidence presented in the literature. Doing it this way allows us to give a more global vision and include those variables related to "what interests us" that cannot be meta-analyzed due to the lack of sufficient cohorts. On the other hand, for quantitative meta-analysis, tables reporting results should include all important information and test statistics, including mean effect sizes, standard error, and confidence intervals. Further, the methods for analyzing the primary outcomes should be the same in all studies to compare results.

**Reviewer comment:** The Discussion does not provide any limitations of the systematic review.

**Author response: **Amended. Limitations have been added.
